# Bioconversion of Pinoresinol Diglucoside and Pinoresinol from Substrates in the Phenylpropanoid Pathway by Resting Cells of *Phomopsis* sp.XP-8

**DOI:** 10.1371/journal.pone.0137066

**Published:** 2015-09-02

**Authors:** Yan Zhang, Junling Shi, Laping Liu, Zhenhong Gao, Jinxin Che, Dongyan Shao, Yanlin Liu

**Affiliations:** 1 College of Food Science and Engineering, Northwest A&F University, 28 Xinong Road, Yangling, Shaanxi Province, 712100, China; 2 Key Laboratory for Space Bioscience and Biotechnology, School of Life Sciences, Northwestern Polytechnical University, 127 Youyi West Road, Xi’an, Shaanxi Province, 710072, China; 3 College of enology, Northwest A&F University, Xinong Road, Yangling, Shaanxi Province, 712100, China; Heidelberg University, GERMANY

## Abstract

Pinoresinol diglucoside (PDG) and pinoresinol (Pin) are normally produced by plant cells via the phenylpropanoid pathway. This study reveals the existence of a related pathway in *Phomopsis* sp. XP-8, a PDG-producing fungal strain isolated from the bark of the Tu-chung tree (*Eucommiaulmoides* Oliv.). After addition of 0.15 g/L glucose to *Phomopsis* sp. XP-8, PDG and Pin formed when phenylalanine, tyrosine, leucine, cinnamic acid, and *p*-coumaric acid were used as the substrates respectively. No PDG formed in the absence of glucose, but Pin was detected after addition of all these substrates except leucine. In all systems in the presence of glucose, production of PDG and/or Pin and the accumulation of phenylalanine, cinnamic acid, or *p*-coumaric acid correlated directly with added substrate in a time- and substrate concentration- dependent manner. After analysis of products produced after addition of each substrate, the mass flow sequence for PDG and Pin biosynthesis was defined as: glucose to phenylalanine, phenylalanine to cinnamic acid, then to *p*-coumaric acid, and finally to Pin or PDG. During the bioconversion, the activities of four key enzymes in the phenylpropanoid pathway were also determined and correlated with accumulation of their corresponding products. PDG production by *Phomopsis* sp. exhibits greater efficiency and cost effectiveness than the currently-used plant-based system and will pave the way for large scale production of PDG and/or Pin for medical applications.

## Introduction

Pinoresinol diglucoside, (+)-1-pinoresinol 4, 4′-di-O-β-D-glucopyranoside ((+)-PDG) is a glycoside lignan compound having various pharmacological functions, including antihypertension [1], [2] and prevention of osteoporosis [3]. After dietary consumption, PDG can be converted by the intestinal microflora to enterolignans [4], which can potentially reduce the risk of breast cancer [5] and other hormone-dependent cancers [6].

Pinoresinol ((+)-Pin) has the strongest anti-inflammatory activity against human intestinal Caco-2 cells among plant lignans [7]. As with PDG, Pin has also been identified as an enterolactone precursor [8] with a preventive effect against breast cancer [9] and endometrial cancer [10]. (+)-Pin is also reported to have a putative hypoglycemic effect though inhibition of α-glucosidase [11].

The demand for lignans (including PDG and Pin) has been rapidly increasing for their capacity to prevent cancer, diabetes, hepatocirrhosis, and hypertension [12]. Tu-chung, *E*. *ulmoides* Oliv., has been the major reported source of PDG in nature [13]. However, the production of PDG from plants is strictly limited by the low content of PDG and the long period needed for tree growth. To our knowledge, only one study is available in the literature by using *Caldariomyces fumage* ATCC 16373 to produce Pin, which is subsequently used to biochemically synthesize PDG [14]. We previously found out *Phomopsis* sp. XP-8 produced PDG, PMG, and Pin during submerged liquid cultivation in defined medium [15] and in the bioconversion system containing whole mung bean or polysaccharides and starch isolated from mung bean [16]. These results indicated a possible way to produce Pin and PDG in dependence of plant growth. However, the biosynthesis efficiency is still very low and far away from enough for production of PDG and Pin in scale. Therefore, it is necessary to understand the intrinsic mechanisms for this strain to produce PDG and Pin.

**Fig 1:**
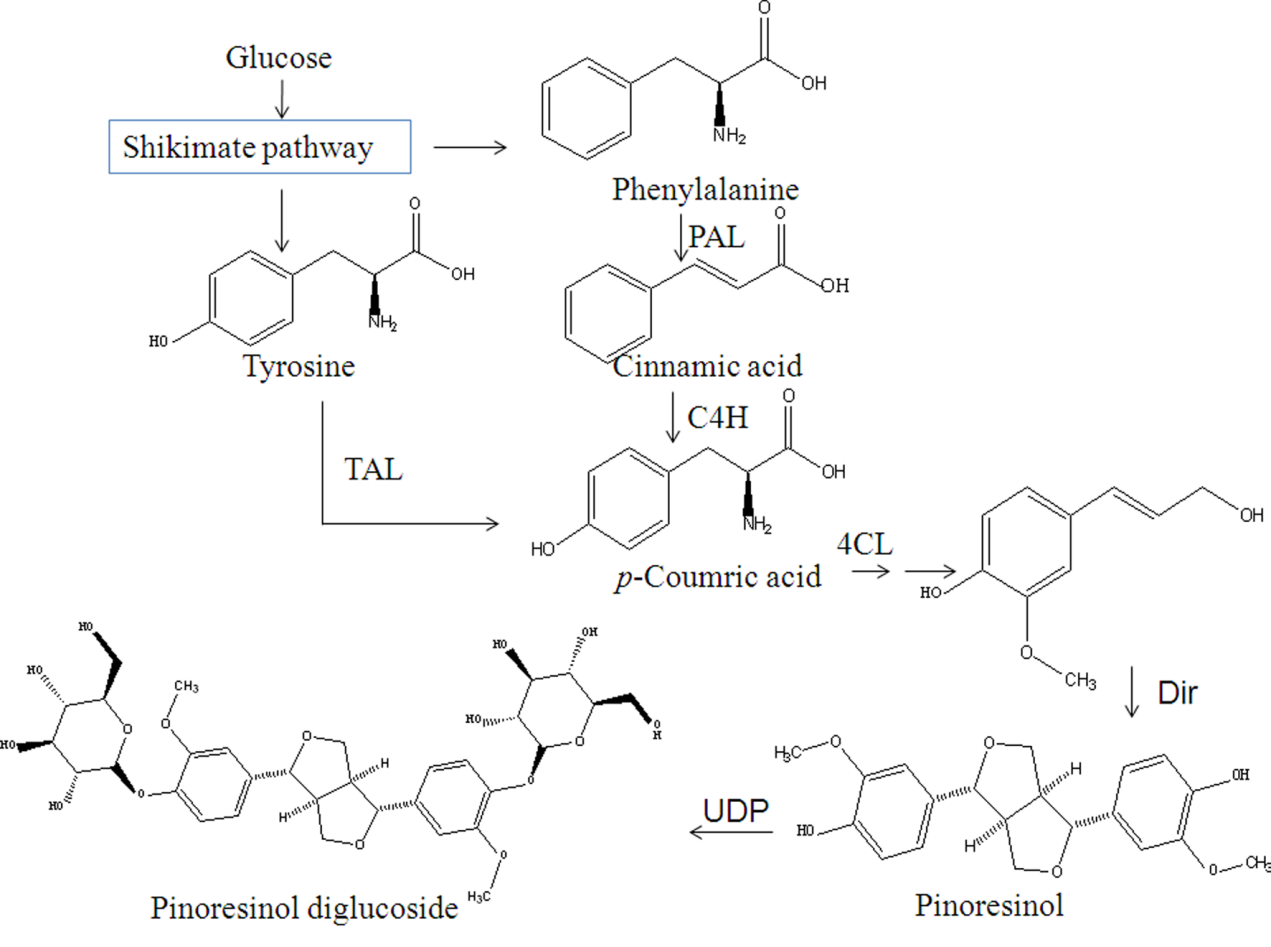
Biosynthetic pathways leading to lignans in plants. Enzymes showed in the figure are phenylalanine ammonia lyase (PAL), tyrosine ammonia lyase (TAL), cinnamate 4-hydroxylase (C4H), and 4-coumaroyl-CoA ligase (4CL), dirigent proteins (Dri) UDP-glucose-dependent glucosyltransferase (UDP)

Biosynthesis of Pin (one of the important natural lignans) and PDG in plants occurs via oxidative coupling of monolignols, which are synthesized through the phenylpropanoid pathway as shown in **Fig 1** [17]. In this pathway, phenylalanine is converted into lignans through many steps with cinnamic acid, *p*-coumarate, *p-*coumaroyl-CoA, caffeate, ferulate, feruloy-CoA, coniferyl aldehyde and coniferyl alcohol as intermediates. Phenylalanine/tyrosine ammonia-lyase (PAL/TAL), trans-cinnamate 4-hydroxylase (C4H) and 4-coumarate-CoA ligase (4CL) are the key enzymes involved in the pathway [18, 19]. Specifically, PAL converts phenylalanine to cinnamic acid, C4H converts cinnamic acid to *p*-coumaric acid and cinnamoyl-CoA to *p*-coumaroyl-CoA, and 4CL, with the aid of ATP and coenzyme A, converts cinnamic acid to cinnamoyl-CoA and *p*-coumaric acid to *p-*coumaroyl-CoA. Finally, *p*-coumaric acid or *p*-coumaroyl-CoA is converted to coniferyl alcohol, which can be converted to Pin by a dirigent protein [20]. Dirigent proteins exhibit unique stereochemical properties and are of great bioorganic chemical interest and are expected to provide a model for biomimetic chemistry and its application [21]. Since PDG is the glycoside of Pin, it was supposed to be converted from Pin by UDP-glucose-dependent glucosyl transferase [12]. However, the biosynthesis pathway of Pin and PDG has not been found in microorganisms, not to mention in *Phomopsis* sp. XP-8.

We previously found *Phomopsis* sp. XP-8 could convert mung bean starch and polysaccharides to Pin, PMG, and PDG. And, the accumulation of phenylalanine, cinnamic acid and *p*-coumaric acid was also detected as products during the process of bioconversion [16]. Therefore, we supposed there might be a similar phenylpropanoid pathway in *Phomopsis* sp. XP-8, and this pathway play an important way in the biosynthesis of Pin and PDG. In order to verify this hypothesis, different substrates and intermediates in the phenylpropanoid pathway were separately feed to *Phomopsis* sp. XP-8 cells in this study, and the accumulation of Pin, PDG, and key components in the phenylpropanoid pathway was determined during the bioconversion. Activities of the key enzymes in the phenylpropanoid pathway were also monitored at the same time. The study is hoped to provide useful information of the biosynthesis pathway of Pin and PDG in *Phomopsis* sp. XP-8, at least in the flow direction of substrates and related enzyme activities.

## Materials and Methods

### Microorganism and reagents

*Phomopsis* sp. XP-8 was used in the study. It was obtained from the China Center for Type Culture Collection (Wuhan, China) (code: *Phomopsis* sp. CCTCC M 209291).

Chromatographically pure leucine (Leu), threonine (Thr), phenylalanine(Phe), glycine (Gly), lysine (Lys), tyrosine (Tyr), tryptophan (Trp), histidine (His), and proline (Pro) (Sigma, St Louis, MO, USA) and cinnamic acid and *p-*coumaric acid (98%; Aladdin, Shanghai, China) were used as substrates in the bioconversion to PDG and Pin. PDG and Pin (≥99%; National Institutes for Food and Drug Control, Beijing, China) were also used as the standards (dissolved by methyl alcohol) in the measurements. CoA-SH (Sigma) and glucose-6-phosphate sodiumsalt (G-6-PNa_2_) and ATP (MP Biomedicals, Santa Ana, CA, USA) were used in the enzyme reactions to detect enzyme activity.

### Preparation of *Phomopsis* sp. XP-8 resting cells and bioconversion conditions

*Phomopsis* sp. XP-8 was prepared as resting cells from a 4-days-old liquid culture in potato dextrose medium and used for the bioconversion of Pin and PDG as that described in detail previously [16]. Unless specifically indicated, the bioconversion was carried out in distilled water (pH 7.0) containing 0.15 g/L glucose (as glycoside supplier) and 10 g cells (wet weight) per 100-mL water solution (as the catelyzer). Conditions for the bioconversion were 48 h at 28°C and 180 rpm (in a 250-mL flask). Besides of glucose and phosphate buffer, different substrates were also added in the bioconversion medium according to the following experimental design.

### Bioconversion of PDG and Pin from different amino acids

Different amino acids (7mmol/L) were individually added in the bioconversion system containing 0.15 g/L glucose and 10 g *Phomopsis* sp. XP-8 cells, and PDG and Pin production was analyzed after the bioconversion. The amino acids used were the aliphatic amino acids leucine (Leu), threonine (Thr), lysine (Lys), the aromatic amino acids phenylalanine (Phe) and tyrosine (Tyr), and the heterocyclic amino acids tryptophan (Trp) and histidine (His).

Three controls were made to eliminate the influence caused by different backgrounds. One was the bioconversion carried out in a medium without any amino acids, but containing glucose and *Phomopsis* sp. XP-8 cells, to make a blank control for the addition of amino acids. Another was the bioconversions in a medium without glucose, but with individual amino acids (7.0 mmol/L) and *Phomopsis* sp. XP-8 cells, to make a blank control for the bioconversion from glucose. The third one was the bioconversion in a medium only containing *Phomopsis* sp. XP-8 cells to make a blank control for the presence of glucose and amino acids.

Each treatment was conducted in three replications and mean values and standard deviations are presented.

### Measurement and identification of accumulated products and intermediates during bioconversion

Accumulation of Phe, cinnamic acid, *p*-coumaric acid, PDG and Pin in the cell-free liquid phase were determined using High Performance Liquid Chromatography (HPLC) [16] and identified using electrospray ionization mass spectrometry (ESI^−^) as described in detail previously [15, 22].

### Intermediates accumulated during bioconversion of PDG and Pin from different amino acids

In the bioconversion medium containing 0.15 g/L glucose and *Phomopsis sp*. XP-8 cells, 7 mmol/L Phe, 7 mmol/L Tyr and 13 mmol/L Leu were individually added to start the bioconversion. During bioconversion, accumulation of cinnamic acid, *p*-coumaric acid, PDG and Pin was monitored at 4, 8, 12, 16, 24, 32, 40, 48, 52 and 64 h when Phe and Tyr were used as the substrate and at 8, 16, 24, 32, 40, 48, 56, 64, 72 and 80 h when Leu was used as the substrate. The effects of different concentration level of the three amino acids were also studied, separately. The levels were 1, 3, 5, 7, 9, 11 and 13 mmol/L for Phe and Tyr, and 1, 3, 5, 7, 9, 11, 13, 15 and 17mmol/L for Leu. After bioconversion for 40, 48, and 56 h when Phe, Tyr, and Leu were used as the substrates, respectively, the accumulation of cinnamic acid, *p*-coumaric acid, PDG and Pin was determined. Each treatment was conducted in three replications. The mean values are presented with standard deviation.

### Bioconversion of PDG and Pin from different substrates in the phenylpropanoid pathway

In the bioconversion system containing glucose and *Phomopsis* sp. XP-8 cells, Phe, cinnamic acid, and *p*-coumaric acid were individually added at different levels to start the bioconversion. The levels were 1, 3, 5, 7, 9, 11 and 13 mmol/L for Phe, and 0.5, 1.0, 1.5, 2.0, and 2.5 mmol/L for cinnamic acid and *p*-coumaric acid. After 40, 32, and 24 h for Phe, cinnamic acid, and *p*-coumaric acid as the substrates, respectively, the accumulation of cinnamic acid and *p*-coumaric acid inside and outside cells was separately detected, and accumulation of PDG and Pin outside cells in the bioconversion system was determined.

Two different controls were made. One was the bioconversion in a medium without glucose containing only *Phomopsis* sp. XP-8 cells. Another one was the bioconversion in a medium without glucose, but with individual addition of Phe, cinnamic acid, or *p*-coumaric acid at different levels. Each treatment was conducted in triplicates.

### Production of different intermediates in the phenylpropanoid pathway and PDG and Pin during bioconversion of PDG and Pin from glucose

In order to understand the biosynthesis pathway of PDG and Pin using glucose as the only starter, bioconversion was also carried out in the system containing only glucose water solution and *Phomopsis* sp. XP-8 cells. Glucose was added at different levels of 5, 10, 15, 20, 25, 30, and 35 g/L. During bioconversion, accumulation of PDG, Pin, Phe, cinnamic acid, and *p*-coumaric acid was monitored every 4 h, up to 56 h.

### Extraction of key enzymes in the phenylpropanoid pathway and measurement of their activities

Key enzymes in the phenylpropanoid pathway were extracted from the cells using phosphate buffer (pH 7.0) and collected after precipitation with ammonium sulfate at 75% saturation according to the method previously reported in detail by Zhang et al [22]. The prepared enzyme was dissolved in 0.2 mol/L phosphate buffer (pH 7.0) and ready for activity measurement.

Measurement of the key enzyme activities was carried out using the method detailed previously [16] and expressed as the activity of per milligram protein. In brief, PAL/TAL activity was measured at 37°C in Tris–HCl buffer (pH 8.9) by following cinnamic acid formation at 290 nm. TAL activity was similarly measured according to the *p*-coumaric acid production at 315 nm using tyrosine as the substrate in the same buffer containing tyrosine. C4H activity was assayed at 340 nm and 30°C in Tris–HCl buffer (pH 8.9) containing cinnamic acid and NADPNa_2_.4CL activity was measured at 333 nm in Tris–HCl buffer (pH 8.9) containing *p*-coumaric acid, CoA-SH, and ATP [16]. Protein concentration was determined according to Bradford techniques [23]. One enzyme unit (U) was defined as an increase of 0.01 of OD value per hour, and the enzyme activity was expressed as the enzyme unit per milligram protein (U/mg) [16].

Each treatment was conducted in three replications. The mean values are presented with standard deviation.

### Activity of key enzymes during bioconversion with different amino acids as substrates

When bioconversion of PDG and Pin using Phe (7 mmol/L), Tyr (7 mmol/L), and Leu (13 mmol/L) as the substrates respectively, the activities of PAL, TAL, C4H, and 4CL of *Phomopsis* sp. XP-8 cells were measured at 4, 8, 16, 24, 32, 40, 48, 52 and 64 h. The effects of amino acids concentration on the enzyme activities of PAL, TAL, C4H, and 4CL were also investigated after 40 h at substrate concentration of 1, 3, 5, 7, 9, 11 and 13 mmol/L for Phe, after 48 h at substrate concentration of 1, 3, 5, 7, 9, 11 and 13 mmol/L forjavascript:void(0); Tyr, after 56 h at substrate concentration of 1, 3, 5, 7, 9, 11, 13, 15 and 17 mmol/L for Leu. Each treatment was conducted in three replications.

### Bioconversion of PDG and Pin from glucose

In the bioconversion medium containing *Phomopsis sp*. XP-8 cells, 20 g/L glucose was added to start the bioconversion. During bioconversion, accumulation of cinnamic acid, *p*-coumaric acid, PDG and Pin was monitored at 8, 12, 16, 24, 32, 40, 48, and 56 h. The effects of different concentration level of glucose were also studied. The levels were 0, 5, 10, 15, 20, 25, 30, and 35 g/L. After bioconversion 48 h, the accumulation of cinnamic acid, *p*-coumaric acid, PDG and Pin was determined. Each treatment was conducted in three replications. The mean values are presented with standard deviation.

## Results

### Bioconversion of PDG and Pin using different amino acids in the presence and absence of glucose

As shown in **Table 1**, biosynthesis of PDG were not found and only little Pin production were found when there was only 0.15 g/L of glucose in the medium (Blank control in **Table 1**). When different amino acids were supplied together with glucose, biosynthesis of Pin and PDG was found only when Phe and tyrosine (Tyr) and leucine (Leu) were used (**Table 1**). Especially, PDG and Pin production was significantly improved when Leu was added (**Table 1**). However, in the absence of glucose, only biosynthesis of Pin was found only when Phe and tyrosine (Tyr) were used as the sole substrates (**Table 2**). This was consistent with the biosynthesis pathway reported in plants, Pin and PDG are synthesized via the phenylpropanoid pathway from Phe or Tyr. It was deduced that Phe and Tyr acted as phenyl donors in the biosynthesis of PDG and Pin. However, Leu does not directly participate in the ligin/lignan metabolic pathway, including the phenylpropanoid pathway [24]. Therefore, we suspected that the improvement of PDG and Pin production by Leu might be due to it indirectly stimulated some kind of key enzyme activities related to the biosynthesis of Pin and PDG.

**Table 1:** Production of PDG and Pin by *Phomopsis* sp. XP-8 cells using different amino acids. Values are the means of three replications and shown with standard deviation.

	Control	Amino acids (7.0 mmol/L) added in the control						
		Leu	Thr	Lys	Phe	Tyr	Trp	His
Dry cell weight (g/L)	1.29±0.15^c^	1.58±0.12^b^ ^c^	1.54 ±0.16^b^ ^c^	1.61 ±0.10^b^ ^c^	2.12 ±0.15^a^	2.01±0.15^a^	1.89±0.12^a^ ^b^	2.18 ±0.14^a^
Pinoresinoldiglucoside (mg/L)	0^d^	1.89±0.14^c^	0^d^	0^d^	3.80±0.16^b^	7.04±0.25^a^	0^d^	0^d^
Pinoresinol (mg/L)	0.59±0.20^d^	5.83±0.20^b^	0^e^	0^e^	13.20±0.25^a^	2.10±0.25^c^	0^e^	0^e^

^a, b, c, d, e^: Different letters in a same raw indicate the data are significantly different as evaluated by Tukey test (p<0.01). The bioconversion medium is sterilized water containing 0.15 g/L glucose. The data were obtained after bioconversion for 40 h. The abbreviations are leucine (Leu), threonine (Thr), lysine (Lys), the aromatic amino acids phenylalanine (Phe) and tyrosine (Tyr), and the heterocyclic amino acids tryptophan (Trp) and histidine (His).

**Table 2:** Production of PDG and Pin by *Phomopsis* sp. XP-8 cells using different amino acids in the absence of glucose. Values are the means of three replications and shown with standard deviation.

	Control	Amino acids (7.0 mmol/L) added in the control						
		Leu	Thr	Lys	Phe	Tyr	Trp	His
Dry cell weight (g/L)	1.02±0.12^d^	1.21±0.10^c^ ^d^	1.13 ±0.12^d^	1.31 ±0.14^b^ ^c^ ^d^	1.78 ±0.12^a^	1.64±0.12^a^ ^b^	1.56±0.14^abc^	1.82±0.14^a^
Pinoresinol (mg/L)	0^c^	0^c^	0^c^	0^c^	10.02±0.4^a^	2.92±0.22^b^	0^c^	0^c^

^a, b, c, d^: Different letters in a same raw indicate the data are significantly different as evaluated by Tukey test (p<0.01). The bioconversion medium is sterilized water without glucose addition. The data were obtained after bioconversion for 40 h. The abbreviations are leucine (Leu), threonine (Thr), lysine (Lys), the aromatic amino acids phenylalanine (Phe) and tyrosine (Tyr), and the heterocyclic amino acids tryptophan (Trp) and histidine (His).

Comparatively, Phe resulted in the highest production of Pin (13.20 mg/L), followed by Leu (5.83 mg/L) and Tyr (2.10 mg/L). Tyr resulted in the highest production of PDG (7.04 mg/L), followed by Phe (3.80 mg/L) and Leu (1.89 mg/L). The production of Pin was much higher than that of PDG by factors of 3.4, and 3.2-fold, respectively when Phe, and Leu were used, while the production of PDG was much higher than that of Pin when Tyr was used (**Table 1**).

Biosynthesis of PDG and Pin was not found when there was only 0.15 g/L glucose in the bioconversion system. However, production of Pin was still observed when Phe and Tyr were used in the medium without glucose. This indicated that the presence of glucose at such low level might mainly act as a glycosyl donor and energy supplier in this case.

In all cases, addition of amino acid substrates tryptophan (Trp), histidine (His), lysine (Lys) and threonine (Thr) did not result in biosynthesis of PDG and Pin, indicating they were not involved in the biosynthetic pathways for PDG and Pin or other pathways related to intermediates involved in these pathways.

It should also be mentioned that, compared with controls without amino acid addition, the addition of amino acids resulted in increase of cell weight after bioconversion in the presence and absence of glucose (**Table 1** and **Table 2**). Comparatively, the addition of His, Phe, Tyr, and Trp resulted in relative higher increase of cell weight than the addition of Lys, Leu, and Thr, in decreasing order.

### Accumulation of different intermediates during bioconversion of PDG and Pin from phenylalanine in the presence of glucose

**Fig 2:**
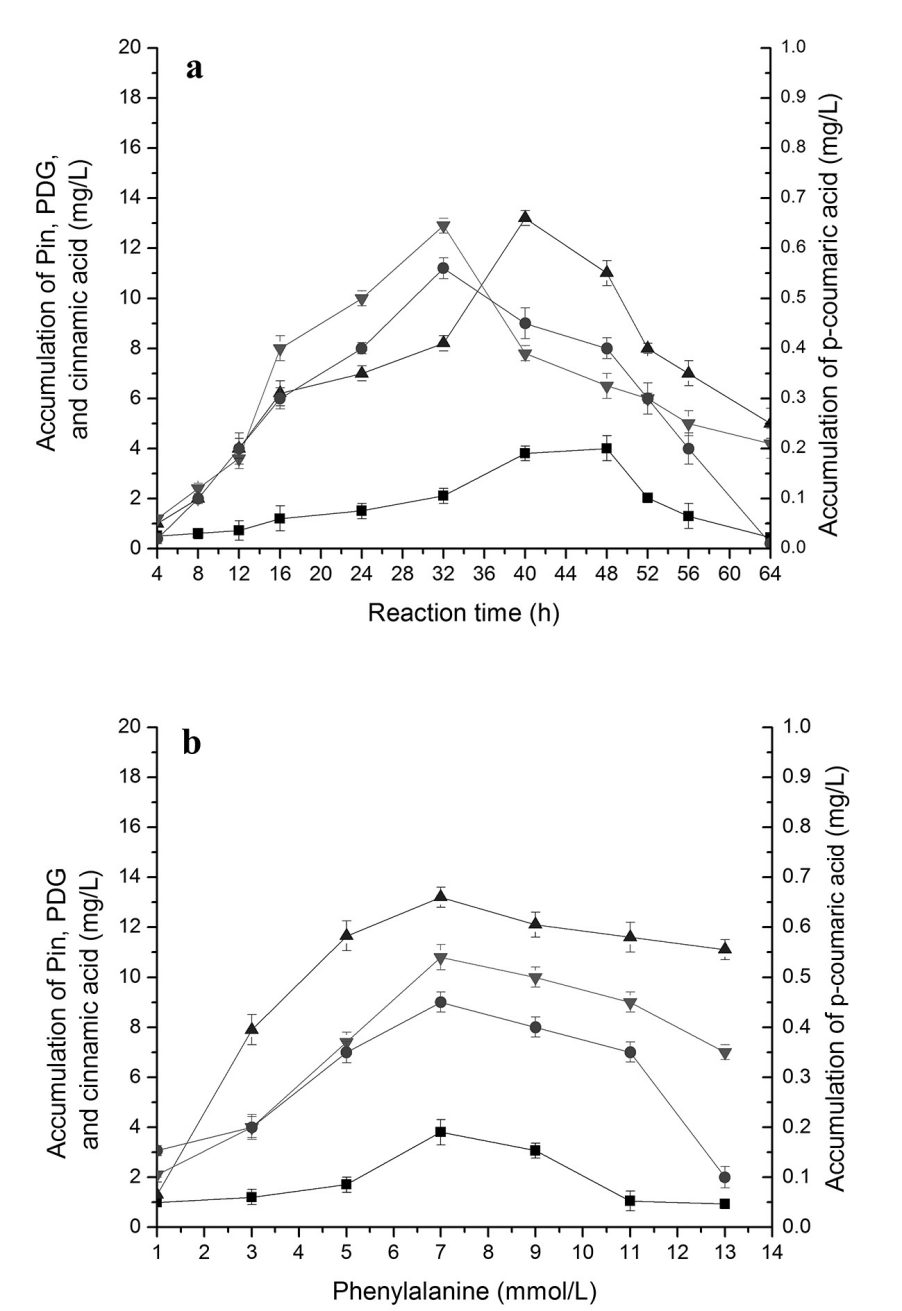
The accumulation of cinnamic acid, *p-*coumaric acid, Pin and PDG during reaction in the resting cell system with phenylalanine as the substrate. The used condition is 7 mmol/L phenylalanine in (a) and reaction time of 40 h in (b). The signals in the figure indicate cinnamic acid (downtriangle), *p-*coumaric acid (circle), Pin (uptriangle), PDG (square).

Bioconversion of cinnamic acid (Ca) from phenylalanine by PAL is the first step of phenylpropanoid pathway [25]. When phenylalanine was used as the only substrate, together with 0.15 g/L glucose, the accumulation of cinnamic acid and *p*-coumaric acid was also found, besides of the production of PDG and Pin, Accumulation of cinnamic acid was much higher than that of *p*-coumaric acid, being consistent with fewer reaction steps from Phe to cinnamic acid in the phenylpropanoid pathway and indicating a higher conversion efficiency of Phe to cinnamic acid (**Fig 2A**). The amounts of cinnamic acid and *p*-coumaric acid showed synchronous changes during bioconversion and similar responses to changes in Phe concentrations (**Fig 2B**).

The highest value of Pin was almost 4-fold higher than that of PDG in all cases, indicating a higher efficiency of Pin production or a smaller number of reaction steps from Phe to Pin than from Phe to PDG. This could be also illustrated by the later appearance of the peak concentration of PDG (48 h) as compared to that of Pin (40 h). Similarly, accumulation of cinnamic acid was almost 20- to 23-fold higher than that of *p*-coumaric acid during the bioconversion. Accumulation of cinnamic acid and *p*-coumaric acid was found when Phe was used as substrate, and accumulation of *p*-coumaric acid was found when cinnamic acid was used as substrate, indicating the reaction flow direction from Phe to cinnamic acid to *p*-coumaric acid. These results are consistent with the reaction flow in the plant phenylpropanoid pathway.

Overall, the accumulations of Pin, PDG, cinnamic acid and *p*-coumaric acid showed similar trend, each increasing early followed by a decrease during the bioconversion and lower yields with increased Phe concentrations (**Fig 2A and 2B**). The highest values for cinnamic acid and *p*-coumaric acid were both obtained at 32 h, for Pin at 40 h and for PDG at 48 h. From the results obtained at 40 h, 7.0 mmol/L Phe appeared to be the optimal Phe concentration for Pin and PDG production and also for the accumulation of cinnamic acid and *p*-coumaric acid.

### Accumulation of different intermediates during bioconversion of PDG and Pin from cinnamic acid and *p-*coumaric acid in the presence of glucose

**Fig 3:**
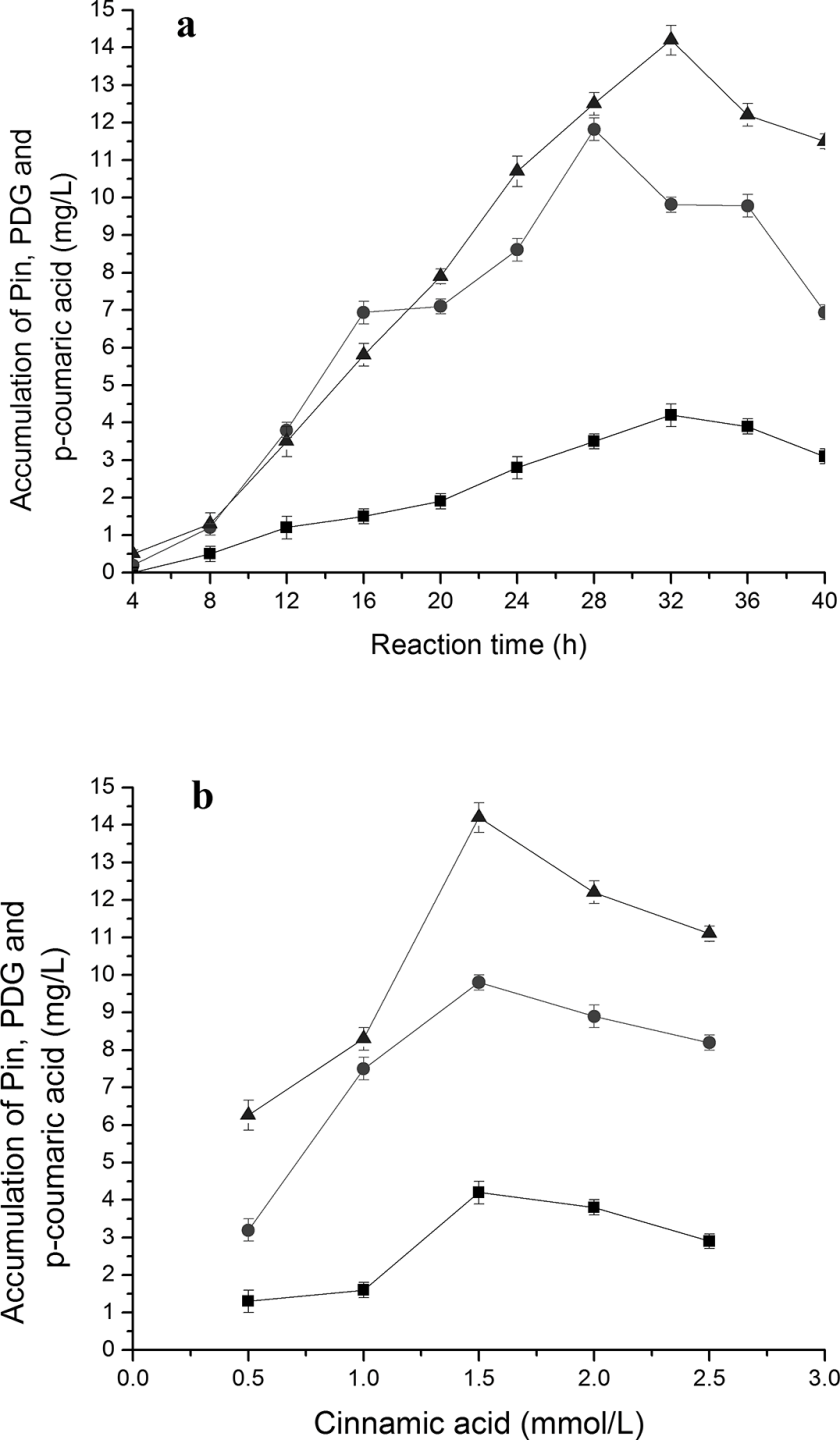
Effects of cinnamic acid additions on *p-*coumaric acid, PDG and Pin production. The signals in the figures indicate PDG (square), Pin (triangle) and *p*-coumaric acid (circle). The used condition is 1.5mmol/L cinnamic acid additions (a) and reaction time of 32 h (b).

**Fig 4:**
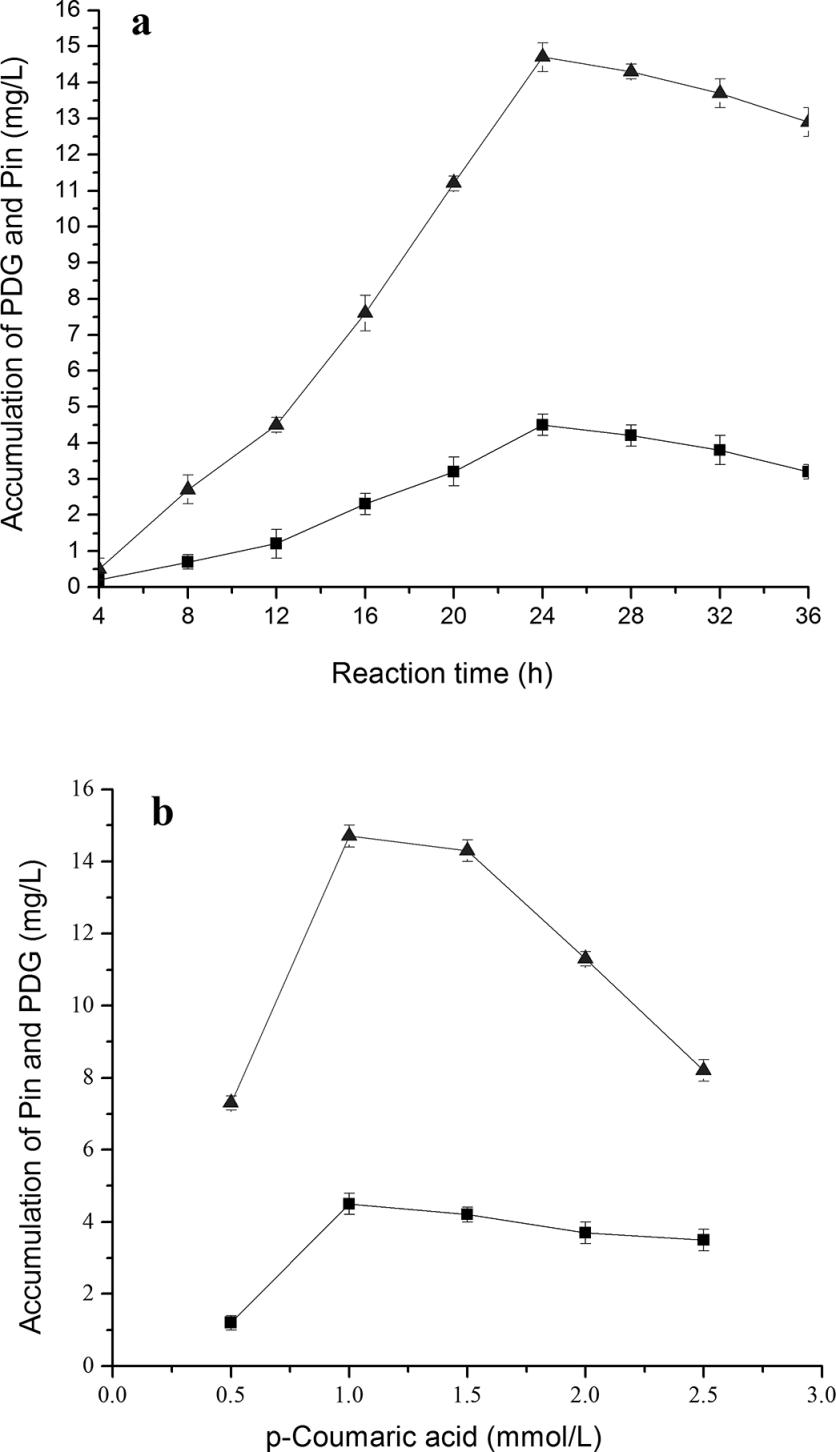
Effects of *p-*coumaric acid additions on PDG and Pin production. The signals in the figures indicate PDG (square), Pin (triangle). The used condition is 1.0 mmol/L cinnamic acid additions (a) and reaction time of 24 h (b).

Production of *p*-coumaric acid, Pin and PDG were found when cinnamic acid (**Fig 3**) was used as the substrate together with 0.15 g/L glucose (as a glycosyl source). Production of PDG and Pin also occurred when *p*-coumaric acid (**Fig 4**) was used as the substrate together with 0.15 g/L glucose (as glycosyl source).

**Fig 3A** shows that when cinnamic acid was used as the substrate, *p*-coumaric acid reached its highest concentration at 28 h, preceding that of PDG (32 h) and Pin (32 h). This result may be due to the presence of fewer reaction steps from cinnamic acid to *p*-coumaric acid than to PDG and Pin. These results are consistent with the substance flow in the plant phenylpropanoid pathway.

The peak production of Pin was about 3.2- to 3.3-fold higher than that of PDG when cinnamic acid and *p*-coumaric acid were used, respectively. Pin production was approximately 4-fold lower when Phe (7 mmol/L) was used. In comparison, use of the substrate *p*-coumaric acid resulted in similar peak values of Pin and PDG (14.71 and 4.52 mg/L, respectively) as found with cinnamic acid (14.21 and 4.20 mg/L). These values were higher than those obtained using phenylalanine (13.20 and 3.82 mg/L).

With the increase of cinnamic acid and *p*-coumaric acid concentrations in medium containing glucose, production of Pin showed a curve increasing early followed by a decrease, indicating the impact of substrate inhibition effects at higher substrate concentrations (**Fig 3B** and **Fig 4B**). Specifically, the highest amount of Pin was observed at 1.5 mmol/L cinnamic acid and 1.0 mmol/L *p*-coumaric acid, lower than the peak point for Phe (7 mmol/L). The highest amount of PDG was observed at 1.5 mmol/L cinnamic acid and 1.0 mmol/L *p*-coumaric acid. Much higher concentrations of PDG and Pin were observed at 1.0 mmol/L *p*-coumaric acid than at 1.5 mmol/L cinnamic acid within 24 h, indicating faster bioconversion of PDG from *p*-coumaric acid than that from cinnamic acid (**Fig 3A** and **Fig 4A**). Similarly, much higher values of Pin and PDG at 1.0 mmol/L *p*-coumaric acid were seen within 24 h than values for 1.0 mmol/L cinnamic acid within 32 h, indicating faster conversion and shorter pathway reaction distance in the bioconversion of Pin and PDG from *p*-coumaric acid than from cinnamic acid. This is also consistent with the reaction flow observed in the plant phenylpropanoid pathway.

### Accumulation of different intermediates during bioconversion of Pin from different substrates in the absence of glucose

**Fig 5:**
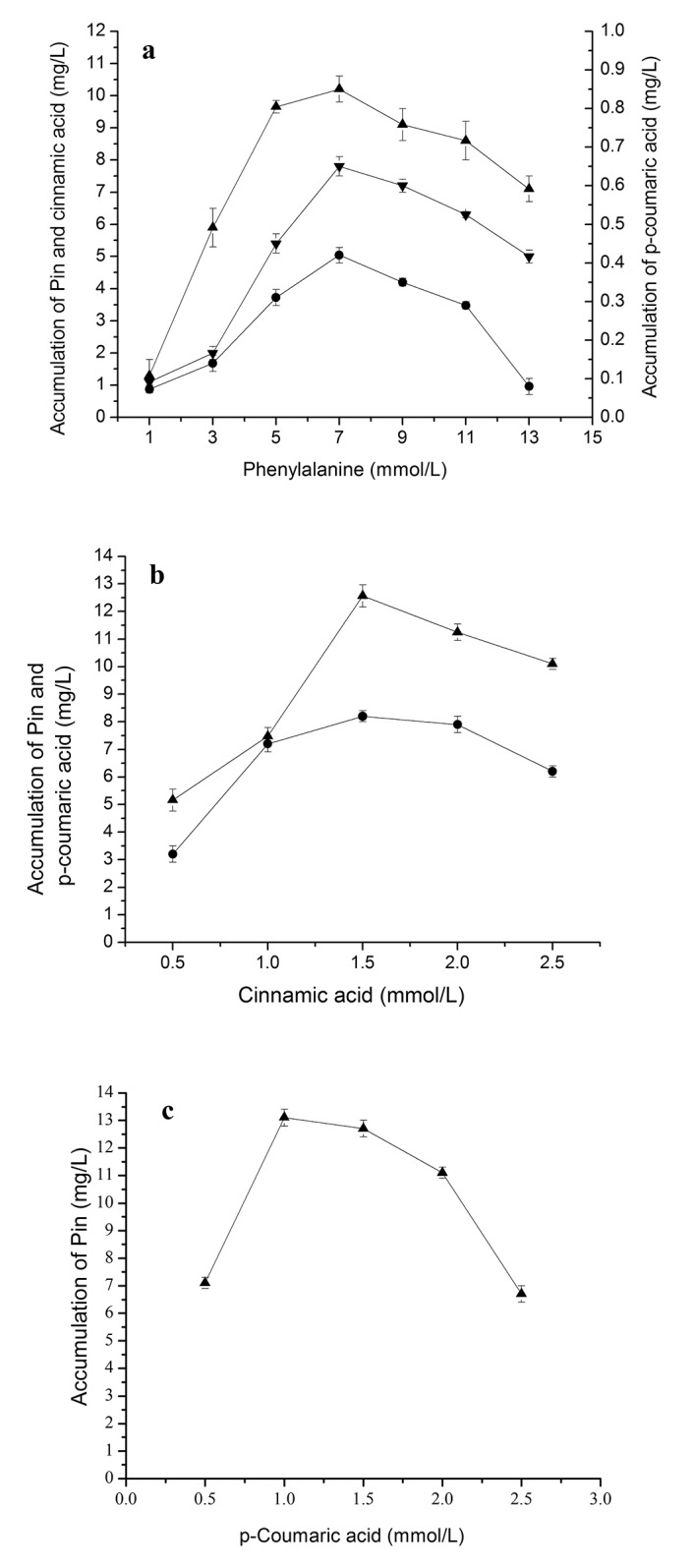
Bioconversion of Pin from Phe, cinnamic acid and *p-*coumaric acid. The used condition is 7 mmol/L phenylalanine within 40 h in (a), 1.5 mmol/L cinnamic acid within 32 h in (b) and 1.0 mmol/L *p*-coumaric acid within 24 h in (c). The signals in the figures indicate cinnamic acid (downtriangle), *p*-coumaric acid (circle), Pin (uptriangle).

Without the presence of glucose in the bioconversion system, no production of PDG was found when Phe, cinnamic acid or *p-*coumaric acid were used separately as the sole substrate (**Fig 5**). However, production of Pin, cinnamic acid and *p*-coumaric acid were found when Phe was used as the sole substrate (**Fig 5A**); Pin and *p*-coumaric acid were found when cinnamic acid was used as the sole substrate (**Fig 5B**); only Pin was found when *p*-coumaric acid was used as the sole substrate (**Fig 5C**). These consisted with the above deduction that glucose might the essential glycol donor for the bioconversion of PDG and the mass flow in the phenylpropanoid pathway.

Production of Pin increased at first and then decreased with the increases of these substrates, indicating the existence of substrate inhibition effects by these compounds on Pin production. Specifically, the highest value of Pin production was observed at 7.0 mmol/L phenylalanine within 40 h (**Fig 5A**), 1.5 mmol/L cinnamic acid within 32 h (**Fig 5B**), and 1.0 mmol/L *p-*coumaric acid within 24 h (**Fig 5C**). Comparing the highest bioconversion rates of different substrates revealed that Pin biosynthesis from *p*-coumaric acid (14.75 mg/L) was similar to that from cinnamic acid (14.27 mg/L) but was higher than that from phenylalanine (13.21 mg/L). This was consistent with the results obtained in the bioconversion system containing 0.15 g/L glucose.

According to the above results, the mass flow related to the bioconversion of Pin and PDG could be deduced as: Phe to cinnamic acid to *p*-coumaric acid. However, it is still not clear which intermediate is the nearest to PDG or Pin in the pathway, and what the relationship is between the bioconversion of Pin and PDG. Furthermore, Pin could not be converted to PDG by *Phomopsis* sp. XP-8, even there was glucose. These results also indicated that the biosynthesis pathway of Pin might be separate from that of PDG in *Phomopsis* sp. XP-8.

### Related enzyme activities during the bioconversion of PDG and Pin from phenylalanine in the presence of glucose

**Fig 6:**
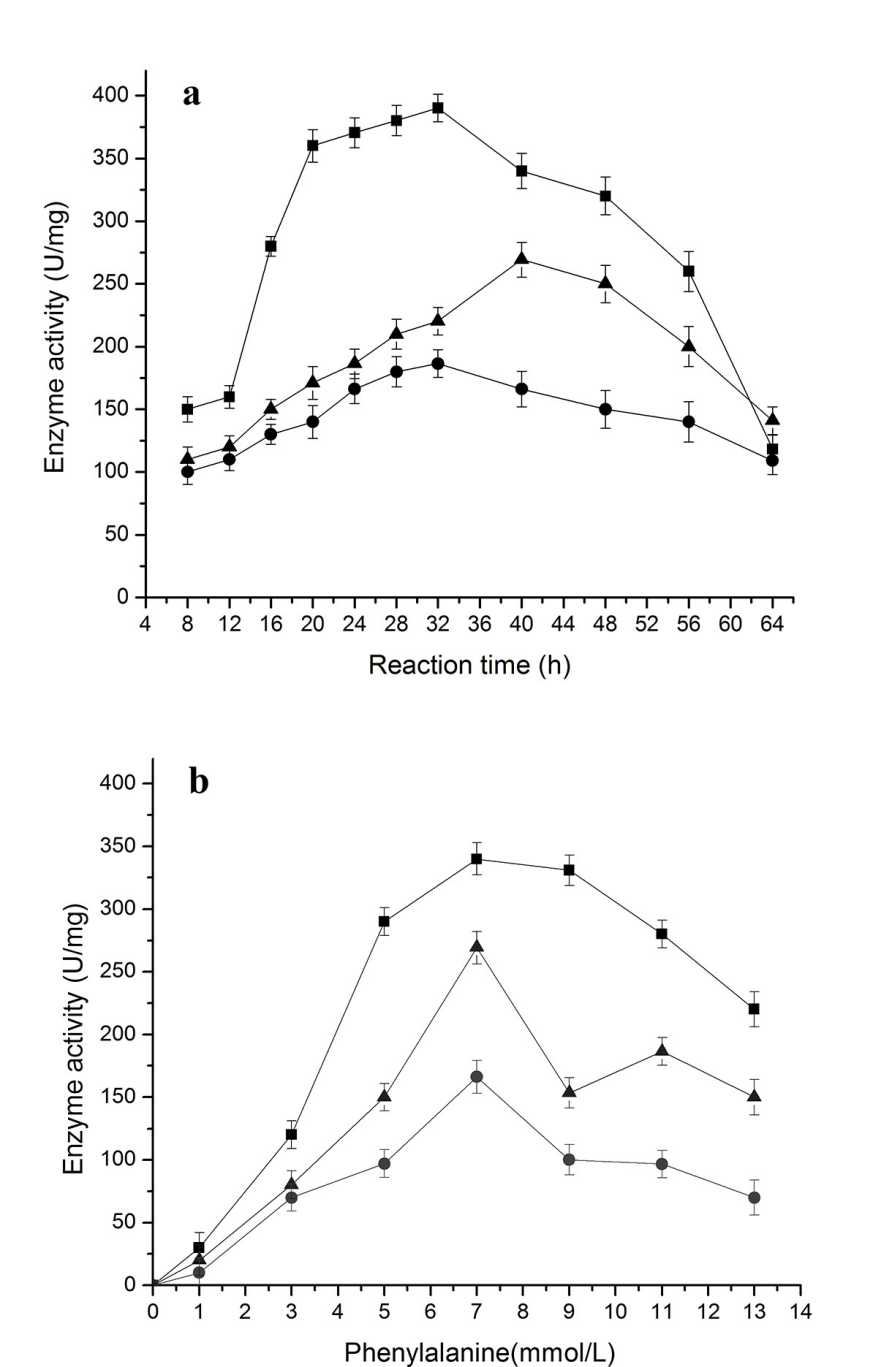
Change of enzyme activities during reaction in the resting cell system with phenylalanine as the substrate. The data were obtained at 7 mmol/L phenylalanine in a, and after 40 h in b. The signals in the figure indicate enzymes 4CL (triangle), C4H (circle), and PAL (square)

When the activities of enzymes PAL, C4H, and 4CL were tracked during the bioconversion, it was found that each enzyme activity showed an increasing curve first, followed by a decrease during the bioconversion and also a decrease with further increase in Phe concentration (**Fig 6**). In all cases, PAL activity was maintained at a higher level than activities of 4CL and C4H, and C4H showed the lowest activity among the three enzymes, being consistent with a much higher accumulation of cinnamic acid than that of *p*-coumaric acid at the same condition (**Fig 2**).

The changes in enzyme activities can be easily rationalized with the patterns of accumulation of intermediates and products throughout the bioconversion process and with the results for different Phe concentrations. Both high PAL activity (converting Phe to cinnamic acid) and low C4H activity (converting cinnamic acid to *p*-coumaric acid) would result in a high accumulation of cinnamic acid. Higher activity of 4CL (converting *p*-coumaric acid to *p*-coumaroyl-CoA) than C4H activity explains the low accumulation of *p*-coumaric acid in all cases.

Over the entire period of bioconversion, the peak value of enzyme activity was obtained at 32 h for PAL and C4H, being coincident with the peak time for the accumulation of cinnamic acid and *p*-coumaric acid, and 40 h for 4CL, being consistent with the peak time of Pin and PDG. The optimal Phe concentration (7.0 mmol/L) for the highest values of all three tested enzyme activities was consistent with that for the highest values of Pin, PDG, cinnamic acid, and *p*-coumaric acid.

### Accumulation of different intermediates and related enzyme activities during bioconversion of PDG and Pin from tyrosine in the presence of glucose

**Fig 7:**
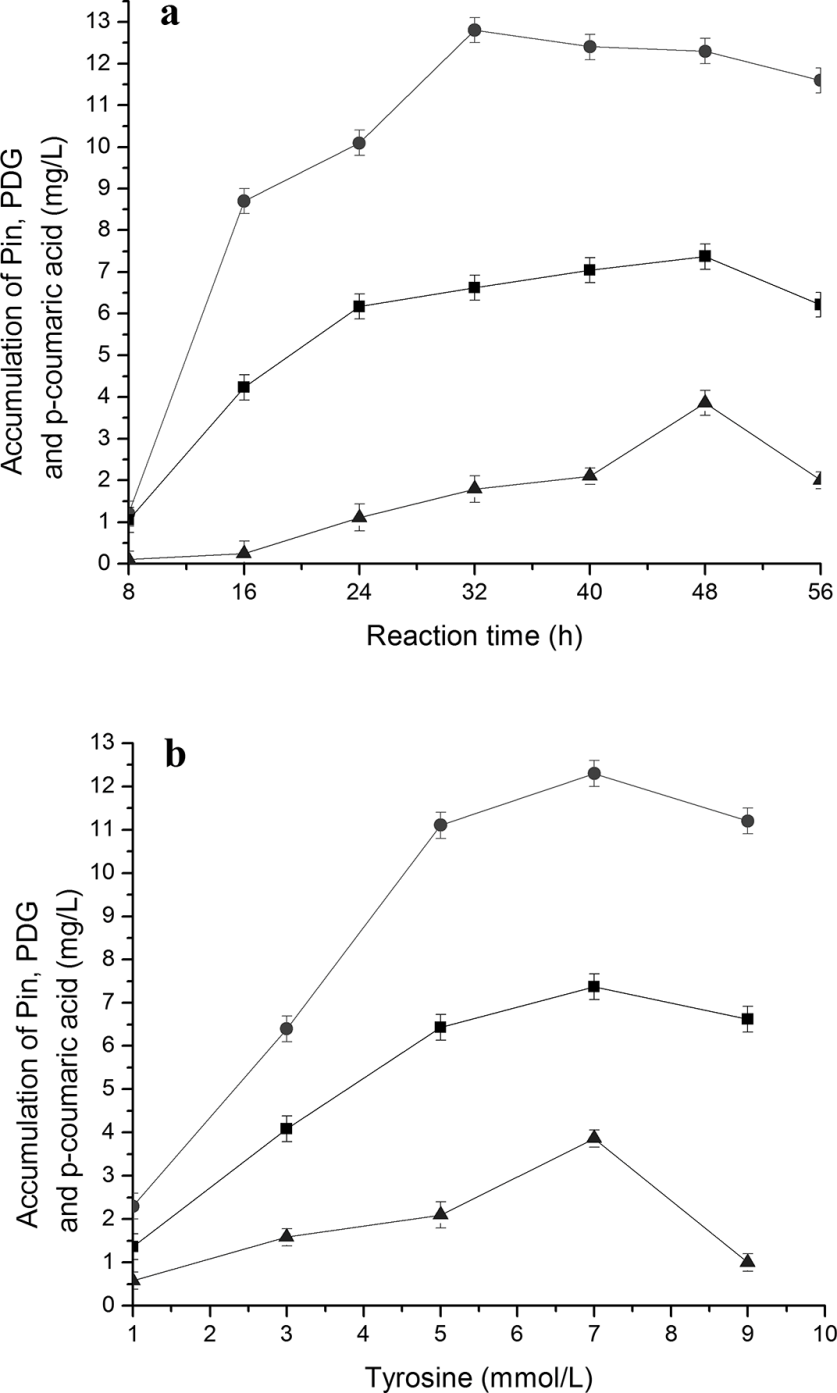
The accumulation of Pin, PDG and *p-*coumric acid during bioconversion in the resting cell system with tyrosine as the substrate. The used condition is 7 mmol/L tyrosine (a) and reaction time of 48 h (b). The signals in the figures indicate PDG (square), Pin (triangle) and *p*-coumaricacid (circle).

Bioconversion of *p*-coumaric acid from tyrosine by TAL is the analagous step to the bioconversion of *p*-coumaric acid from phenylalanine by PAL and C4H in the reported plant phenylpropanoid pathway as shown in **Fig 1** [26]. As expected, accumulation of *p*-coumaric acid, as well as production of PDG and Pin, was found when tyrosine was used as the only substrate together with 0.15 g/L glucose (as a glycosyl supplier). Accumulation of *p*-coumaric acid was much higher by using of tyrosine than using of Phe, indicating fewer reaction steps and higher conversion efficiency of *p*-coumaric acid from tyrosine than from Phe (**Fig 7**). The highest value of PDG was higher than that of Pin when tyrosine was used as the only substrate, indicating higher efficiency of converting Pin to PDG under these conditions.

Overall, the accumulations of Pin, PDG and *p*-coumaric acid showed similar trends with the increase in Tyr concentration, first increasing then decreasing during the bioconversion (**Fig 7B**). The highest value was obtained at 32 h for *p*-coumaric acid, 48 h for Pin and PDG, indicating a faster accumulation of *p*-coumaric acid than that of Pin and PDG. According to the results obtained at 48 h, 7 mmol/L tyrosine appeared to be the optimal tyrosine concentration for production of *p*-coumaric acid, Pin and PDG.

**Fig 8:**
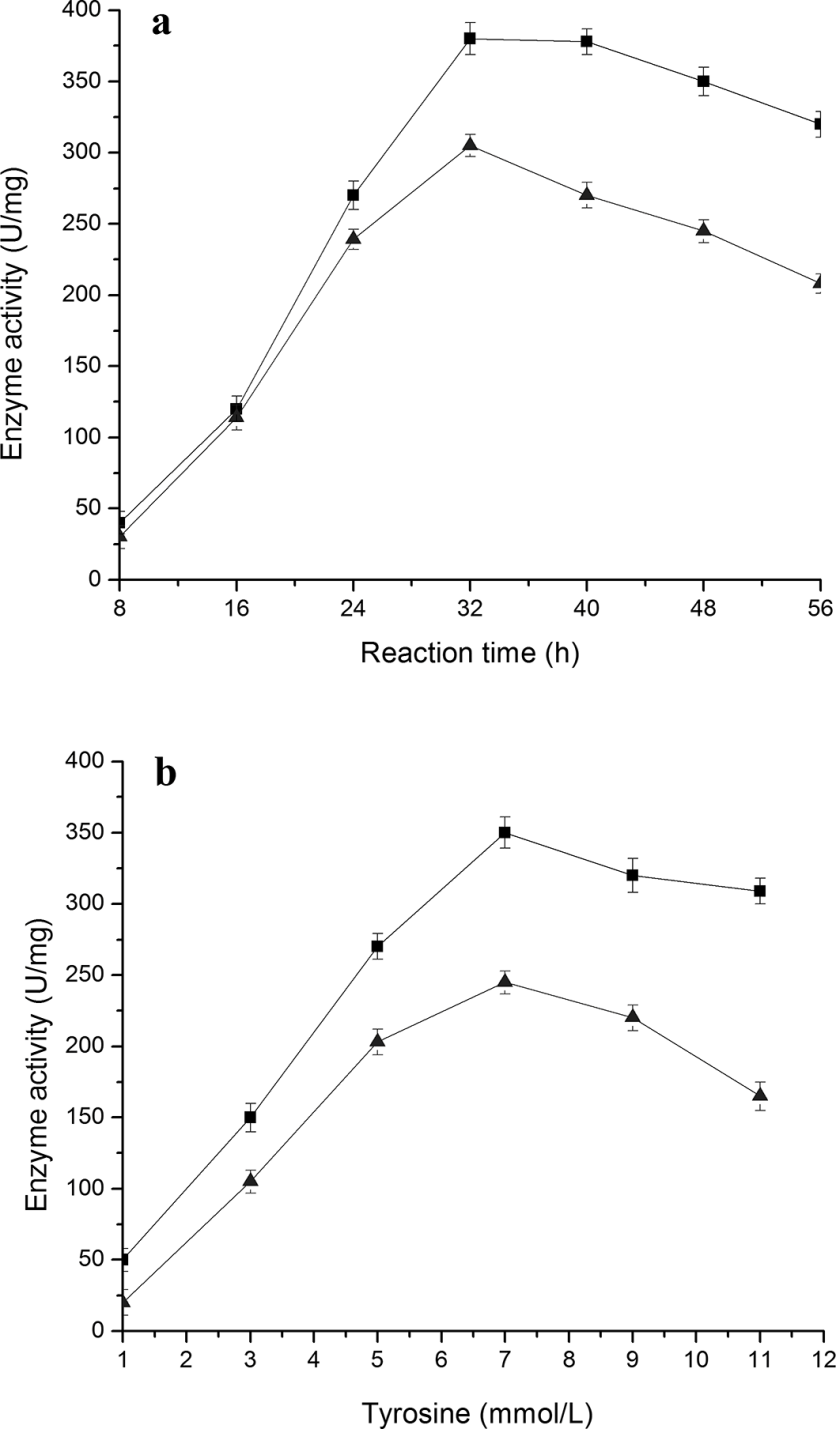
Change of enzyme activities during reaction in the resting cell system with tyrosine as the substrate. The data were obtained at 7 mmol/L tyrosine (a) and after 48 h (b). The signals in the figure indicate enzymes 4CL (triangle) and TAL (square).

When the activities of enzymes TAL and 4CL were tracked during the bioconversion, it was found that each enzyme activity initially showed an increasing curve, followed by a decreasing curve with the increase of tyrosine concentration (**Fig 8B**). TAL and 4CL activities reached the highest point when 7 mmol/L tyrosine was used in the bioconversion medium, which was consistent with the highest accumulation of *p*-coumaric acid, PDG and Pin under these conditions (**Fig 7B**). The TAL and 4CL activities reached the highest point at 32 h (**Fig 8A**), in agreement with the accumulation of *p*-coumaric acid, PDG and Pin under these conditions (**Fig 7A**). This may indicate that a suitable concentration of tyrosine stimulates TAL activity and that high TAL activity results in a high *p*-coumaric acid accumulation. A high concentration of *p*-coumaric acid improves 4CL activity, which causes production of high amounts of PDG and Pin.

The changes in enzyme activities explain the accumulation of intermediates and products during the whole period of bioconversion and can be reconciled to the trends seen with different tyrosine concentrations. Both high TAL activity (converting tyrosine to *p*-coumaric acid) and high 4CL activity (converting *p*-coumaric acid to *p-*coumaroyl-CoA) would result in high accumulation of PDG and Pin.

The highest value of 4CL activity (305 U/mg) obtained at 32 h with 7 mmol/L tyrosine (**Fig 8**) was higher than the highest value of activity (269 U/mg) obtained at 40 h with 7 mmol/L phenylalanine (**Fig 6**). This may result because of the higher accumulation of *p*-coumaric acid in the medium when tyrosine is used as the substrate than when phenylalanine is used. The highest value of *p*-coumaric acid accumulation (12.81 mg/L) obtained at 32 h with 7 mmol/L tyrosine (**Fig 7**) was much higher than the highest value of *p*-coumaric acid accumulation (0.56 mg/L) obtained at 32 h with 7 mmol/L phenylalanine (**Fig 2**). This result can be explained by the low C4H activity (converting cinnamic acid to *p*-coumaric acid) when phenylalanine is used as the substrate (**Fig 6**). There are two steps from phenylalanine to *p*-coumaric acid, while there is only one step from tyrosine to *p*-coumaric acid. The low C4H activity thus may limit the production of *p*-coumaric acid from phenylalanine.

### Accumulation of different intermediates and related enzyme activities during bioconversion of PDG and Pin from leucine in the presence of glucose

**Fig 9:**
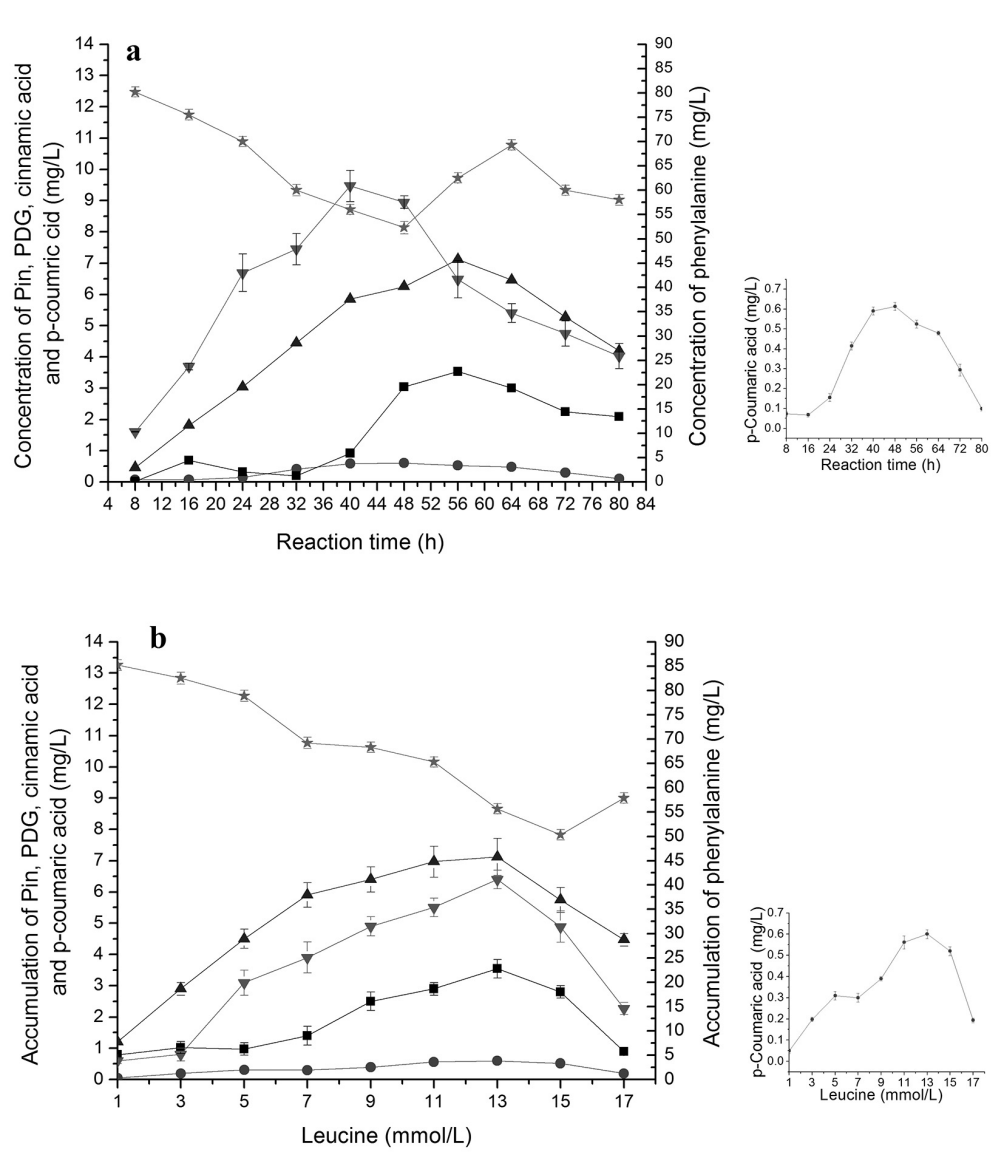
The accumulation of Phe, cinnamic acid, *p-*coumaric acid, Pin and PDG during bioconversion in the resting cell system with leucine as the substrate. The used condition is 13 mmol/L leucine in a and reaction time of 56 h (b). Signals indicate Phe (five-point star), cinnamic acid (downtriangle), *p*-coumaric acid (circle), Pin (uptriangle) and PDG (square).

**Fig 10:**
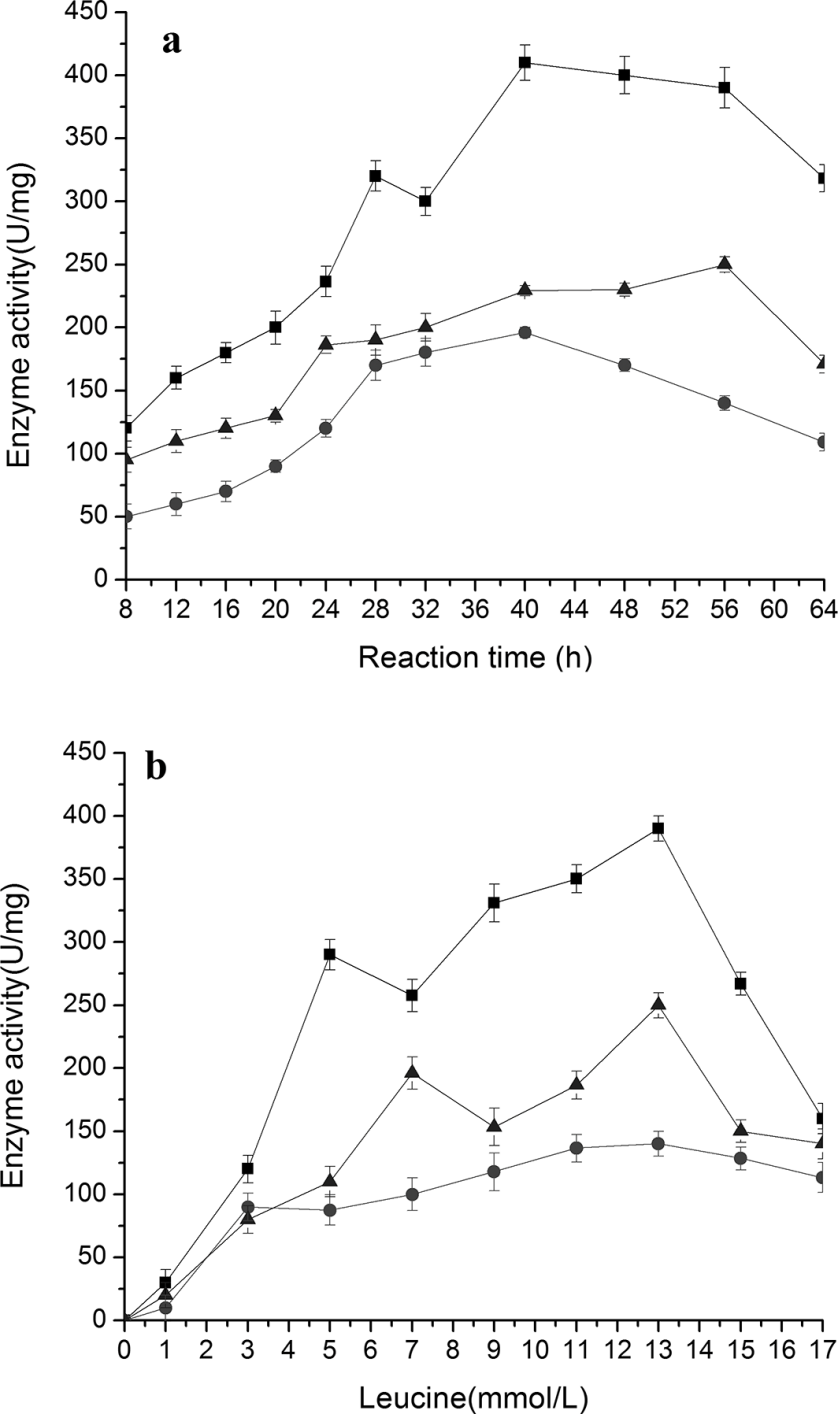
Change of enzyme activities during reaction in the resting cell system with leucine as the substrate. The data were obtained at 13 mmol/L leucine(a) and after 56 h (b). The signals in the figure indicate enzymes 4CL (triangle), C4H (circle), and PAL (square).

Similar to the results when Phe was used, addition of Leu to the bioconversion system containing 0.15 g/L glucose resulted in accumulation of intermediates cinnamic acid and *p*-coumaric acid and the products Pin and PDG (**Fig 9**). Also, the activities of PAL, 4CL, and C4H showed corresponding changes with the bioconversion period and substrate concentration, in order from higher activity to lower activity of PAL, 4CL, and C4H (**Fig 10**).

All detected parameters showed a similar curve which increased at first, followed by a decrease. The peak time was obtained at 40 h for cinnamic acid (9.46 mg/L), 48 h for *p*-coumaric acid (0.61 mg/L),and 56 h for Pin (7.12 mg/L) and PDG (3.53 mg/L) (**Fig 9A**). The highest values of PAL and C4H activity were also obtained at 40 h, still coincident with the peak time of cinnamic acid (**Figs 9A** and **10A**). The peak time of 4CL was obtained at 56 h, being consistent with the peak time of Pin and PDG (**Figs 9A** and **10A**). Also, 13 mmol/L was the optimal Leu concentration to yield the highest accumulation of intermediates and products and the highest relevant enzyme activities (**Figs 9B** and **10B**).

Compared to the results obtained when Phe, cinnamic acid, or *p*-coumaric acid was used as the substrate, the accumulation of cinnamic acid and the production of Pin were much lowered, and the peak time was delayed when Leu was used as the substrate. Except for the delayed peak time, the accumulation of *p*-coumaric acid and the production of PDG were slightly influenced when Leu was used as the substrate, compared with that when Phe was used. In addition, the optimal Leu substrate concentration to produce the highest amounts of intermediates and products (13.0 mmol/L) was higher than that when Phe was used (7.0 mmol/L). All these results indicate that Phe was more efficient than Leu in producing Pin and PDG.

When Leu was used as the sole substrate without glucose addition, no production of cinnamic acid, *p*-coumaric acid, PDG and Pin were observed in the medium. However, Phe was found in the medium when Leu was used as the substrate with 0.15 g/L glucose. We propose that the effect of Leu on improvement of PDG and Pin production may be through stimulation of *Phomopsis* sp. XP-8 to metabolize glucose to produce Phe, resulting in production of PDG and Pin via the phenylpropanoid pathway, since when no Leu was added to the bioconversion medium (only 0.15 g/L glucose alone), no production of PDG, Pin, cinnamic acid and *p*-coumaric acid were found.

### Accumulation of different intermediates during bioconversion of PDG and Pin from glucose

**Fig 11:**
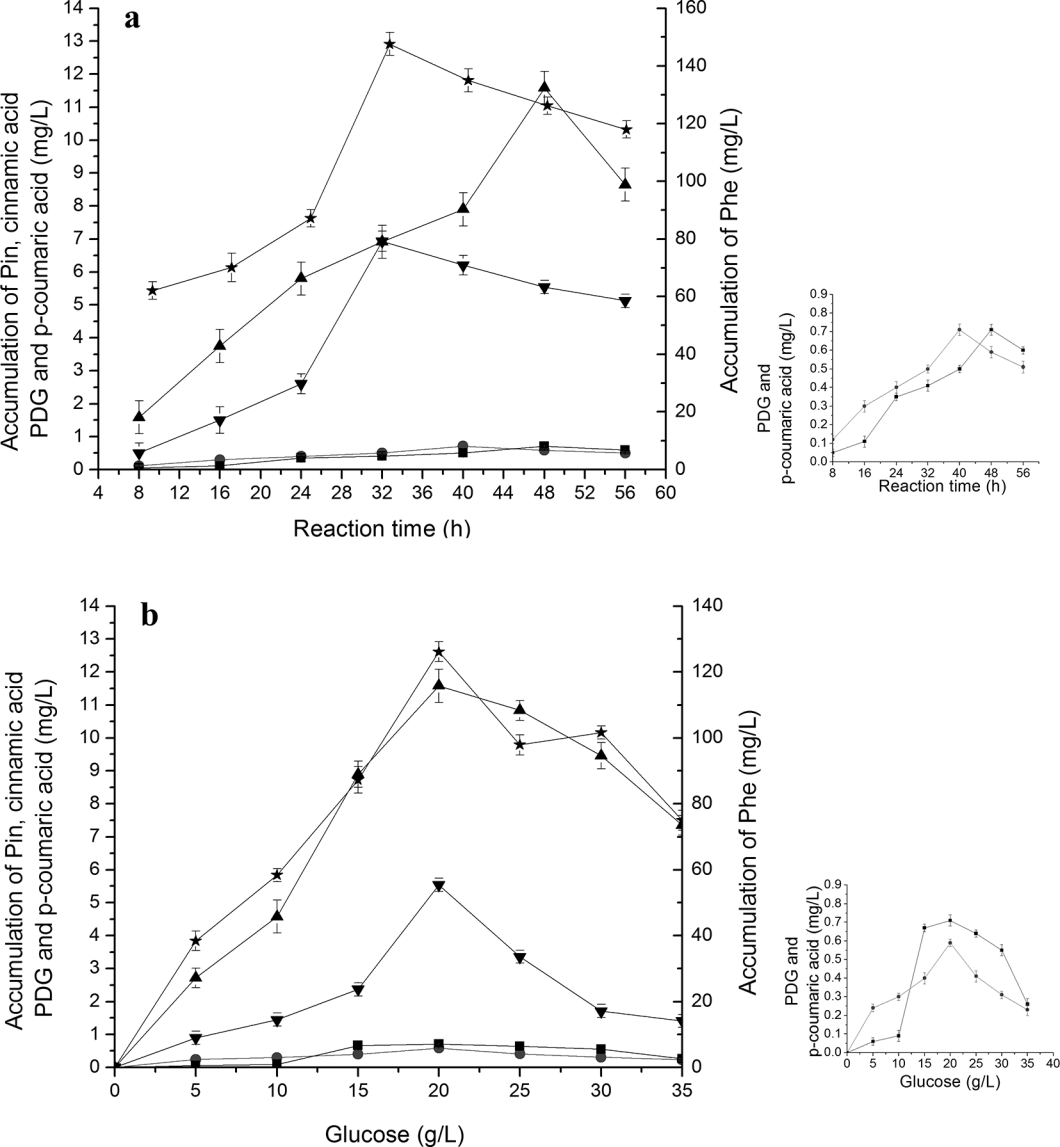
The accumulation of cinnamic acid, *p-*coumaric acid, Pin and PDG during bioconversion in the resting cell system with glucose as the substrate. The used condition is 20 g/L glucose(a) and reaction time of 48 h (b). Signals indicate Phe (star) cinnamic acid (downtriangle), *p*-coumaric acid (circle), Pin (uptriangle) and PDG (square).

When the glucose addition increased from 5 to 35 g/L, production of Pin and PDG showed a first increase followed by a decrease with the increase of glucose concentration addition, and reached a highest value at 20 g/L glucose (**Fig 11B**). This result revealed that *Phomopsis* sp. XP-8 can use glucose as the only substrate to produce PDG and Pin when the glucose concentration was high enough in the conversion medium. The accumulation of Phe, cinnamic acid and *p*-coumaric acid (intermediate substrates of the phenylpropanoid pathway) was also found in the medium when glucose was used as the only substrate, indicating the presence of a phenylpropanoid pathway whereby *Phomopsis* sp. XP-8 utilizes glucose to produce PDG and Pin.

During the bioconversion of glucose, accumulation of PDG and Pin reached the highest point at 48 h, which was longer than the result when Phe was used as the only substrate. This may because that there are more steps from glucose to PDG and Pin than from Phe.

### Efficiency of each point in the mass flow for the bioconversion of PDG or Pin from different substrates

**Fig 12:**
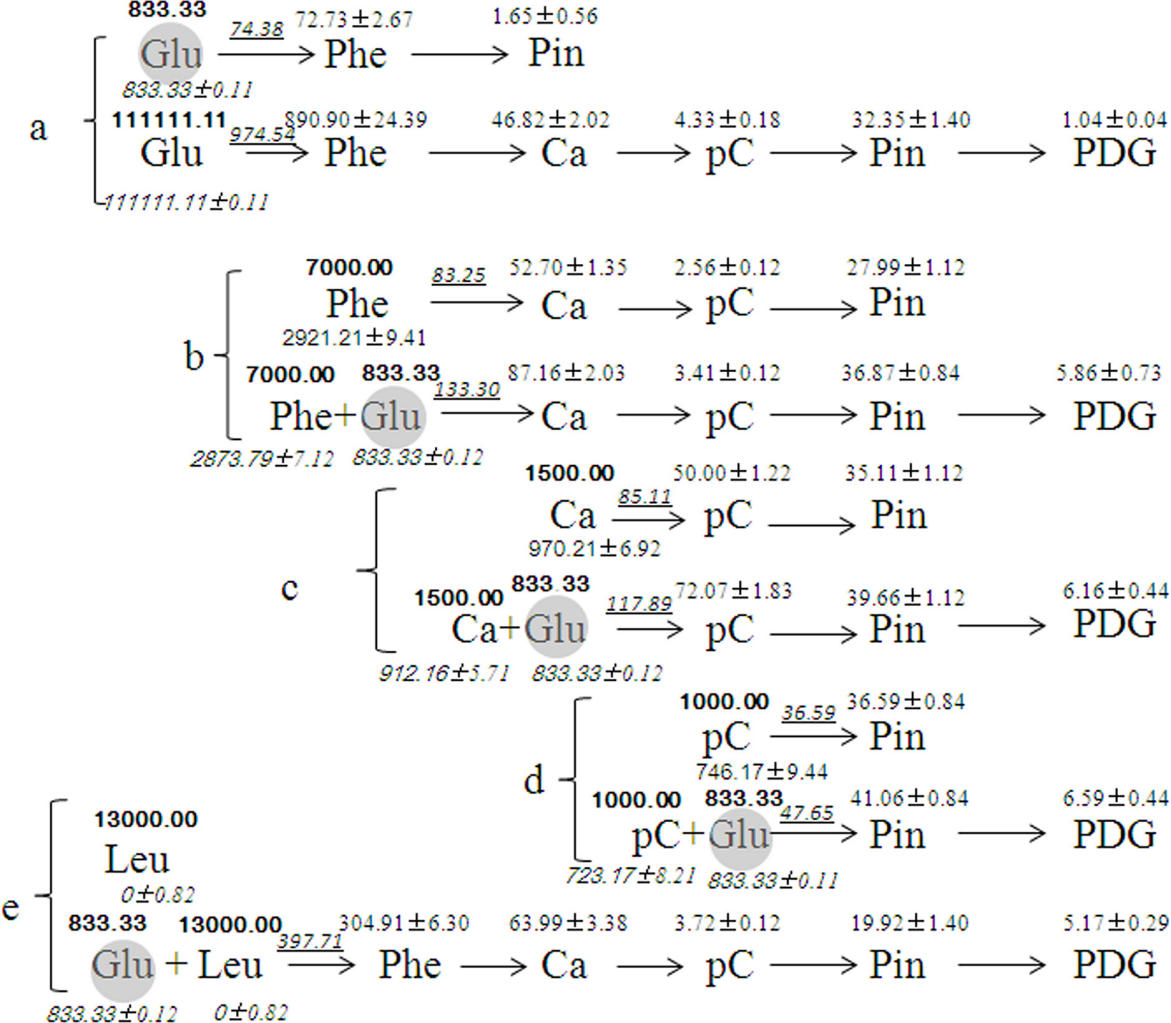
Summary of the mass flow during bioconversion of Pin and/or PDG from glucose (a), phenylalanine (b), cinnamic acid (c), *p*-coumaric acid (d), and leucine (e). The data shown in the figure indicate the initial amount (Arial Bold type) and the consumed amount (Arial italic type) of the substrates, the amount of metabolic flux of the substrates to the presented pathway (Arial italic with underline type) and the highest productions of the substrates (Times normal type). The unit is μmol/L for all present values. Values presented here are the mean of three replications with standard deviation. The values were calculated according to Eq (1).
$$\Delta \text{C}=\frac{\left(\text{C}-{\text{C}}_{0}\right)\times 1000}{M}$$(1)
Where, the letters indicate the corresponding data of each substrate, specifically, △C is the highest amount (μmol/L) of the products during the bioconversion, C is the highest amount (mg/L) after the bioconversion, C_0_ (mg/L) is the initial amount, and M (μg/μmol) is the molecular weight. The abbreviations in the figure mean glucose (Glu), leucine (Leu), phenylalanine (Phe), tyrosine (Tyr), cinnamic acid (Ca), *p*-Coumaric acid (pC), pinoresinol (Pin), and pinoresinol diglucoside (PDG).

The mass flow and the efficiency of each step in the mass flow can be clearly seen in **Fig 12**. When glucose was solely used, the accumulation of PDG and Pin and key intermediates in the phenylpropanoid pathway were found at high glucose concentrations, but only production of Pin was detected at low glucose concentration (833.33 μmol/L or 0.15 g/L) **(Fig 12A)**.

As shown in **Fig 12B–12D,** when different intermediates in the phenylpropanoid pathway were added in the medium, accumulation of key intermediates and production of PDG and Pin were detected, even at low glucose concentration. However, in the absence of glucose, only production of Pin was observed. The optimum concentration of each substrate for the highest productions of PDG and Pin was 111111.11 μmol/L of glucose, 7000.00 μmol/L of Phe, 1500.00 μmol/L of cinnamic acid, and 1000.00 μmol/L of *p*-coumaric acid, following an order that the shorter distance in pathway for the bioconversion of Pin and PDG, the lower concentration substrates was needed.

It should be mentioned that no accumulation of any products was found when Leu was solely used as the substrate (**Fig 12E**). However, the accumulation of all tested intermediates in the phenylpropanoid pathway and production of Pin and PDG were detected when it was used, in the presence of very little glucose (833.33 μmol/L) (**Fig 12E**).

Accumulation of Phe production was 72.73±2.67 μmol/L in the presence of 833.33 μmol/L glucose (**Fig 12A**), while the optimum concentration of Phe to produce PDG and Pin were 7000.00 μmol/L (**Fig 12B**), corresponding to the condition for the highest value of PAL activity (390.00±11.00 (U/mg) (**Fig 6, Table 3**). PAL activity was very low at Phe concentration of 72.73±2.67 μmol/L, explaining the low production of Pin and absence of the accumulation of other intermediates in the phenylpropanoid pathway when 833.33 μmol/L glucose was solely used as the substrate. When Leu was added, together with glucose, the highest value of PAL activity reached 410.00 ± 14.00 U/mg (**Fig 10, Table 3**). This result provided a reasonable explanation for the improvement of production of PDG and Pin by the addition of Leu in the medium containing glucose, that is the presence of Leu increased PAL activity needed for the biosynthesis of PDG and Pin.

**Table 3:** The highest values of each enzyme activity and the occurrence time during the bioconversion with different amino acids as substrates in the presence of glucose. Values are the means of three replications and shown with standard deviation.

Substrate	PAL (U/mg)	Time (h)	TAL (U/mg)	Time (h)	C4H (U/mg)	Time (h)	4CL (U/mg)	Time (h)
Phe	390.0±11.0^a^	32	-		186.3±11.0^a^	32	269.2±13.0^b^	40
Tyr	-	-	380.0±11.0^a^	32	-	-	305.0±8.0^a^	32
Leu	410.0±14.0^a^	40	-	-	196.1±4.0^a^	40	250.0±6.0^b^	56

^a, b^: Different letters in a same column indicate the data are significantly different as evaluated by Tukey test (p<0.01). ‘-’means the results are not detectable. The abbreviations are phenylalanine (Phe), tyrosine (Tyr), leucine (Leu), phenylalanine (PAL), tyrosine ammonia-lyase (TAL), trans-cinnamate 4-hydroxylase (C4H) and 4-coumarate-CoA ligase (4CL).

It was also found that there was only a small amount of the supplied glucose and other substrates flowed into the biosynthesis pathway of Pin and/or PDG, although large amount of them was supplied (**Fig 12**). This might be due to some of the supplied substrates were used for cell growth or other biosynthesis pathways. Further study is needed to increase the bioconversion efficiency by regulating the mass flow among different biosynthesis pathways in the metabolism net.

**Fig 13:**
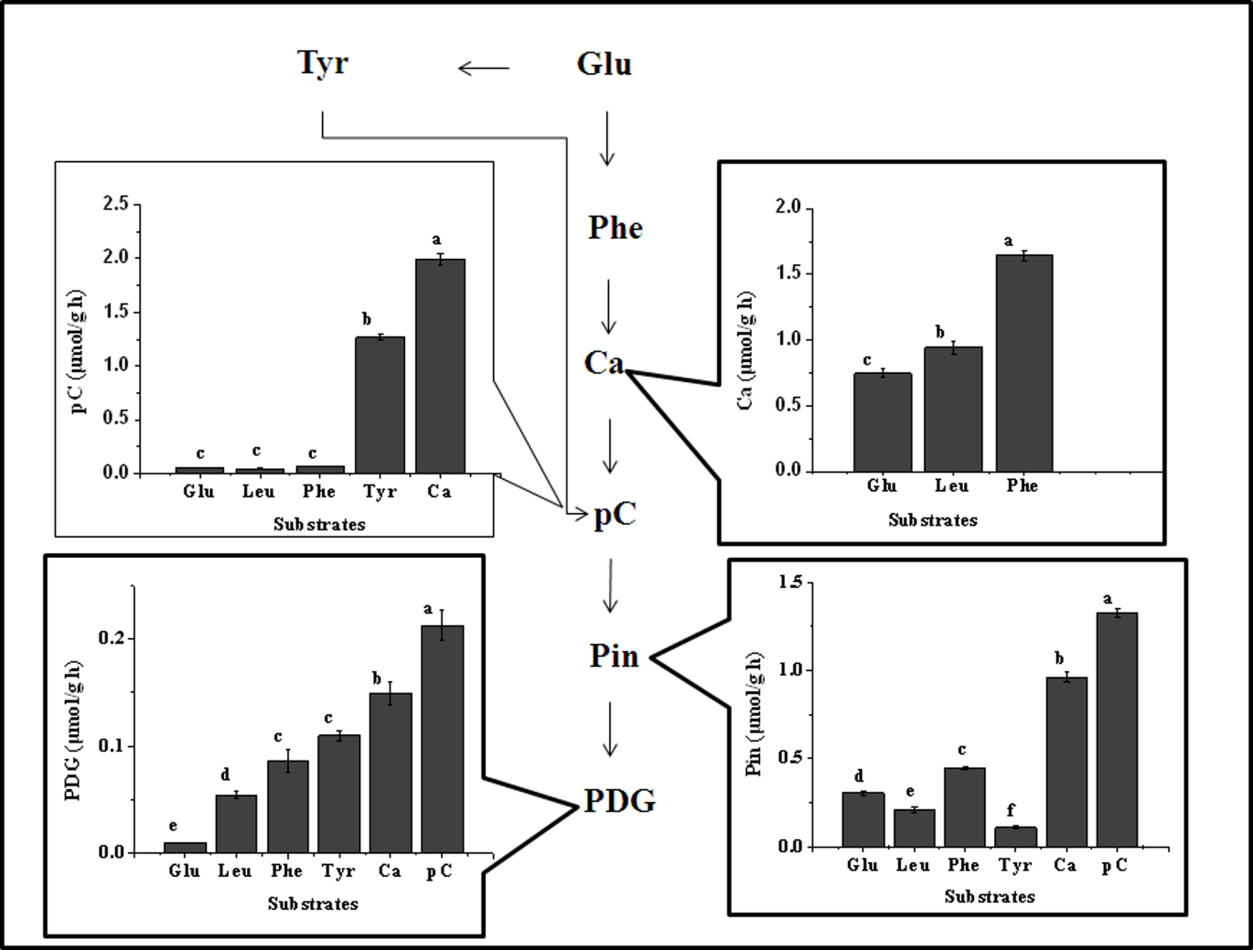
Effect of the addition of different substrates on the accumulation of each intermediate and product. The highest values of the products per g dry cell weight and per hour were calculated by Eq (2).
$$\text{v}=\frac{\Delta \text{C}}{\Delta \text{t}\times \text{X}}$$(2)
where v is the highest value (μmol/g h) of each intermediate or product, △C is the amount of accumulated amount (μmol/L) of the products during the bioconversion, △t is the bioconversion time (h) corresponding to the highest value of each intermediate or product, and X is the amount of cells in dry cell weight (g/L). The amount of the added substrates were 111111.11 μmol/L (Glu), 13000.00 μmol/L (Leu), 7000.00 μmol/L (Phe), 5000.00 μmol/L (Tyr), 1500.00 μmol/L (Ca), and 1000.00 μmol/L (pC) respectively. The bioconversion medium contained 833.33 μmol/L glucose. Values in each figure are the mean of three replications and shown with standard deviation. Different letters in the figure indicate the data were significantly different at the level of p<0.01 in Tukey test.

When cell growth during the process of bioconversion was considered, the accumulation of different intermediates and products per g of dry cell weight and per hour was obtained and shown in **Fig 13.** It can be clearly seen that the biosynthesis efficiency of each intermediate or product was highest when its direct precursor was used, but less when indirect precursors were used. This confirmed the above deduced mass flow in the biosynthesis of Pin and PDG in *Phomopsis* sp. XP-8.

## Discussion

The study provided some preliminary, but essential and important evidences on the occurrence of phenylpropanoid metabolism in microorganisms. The phenylpropanoid pathway in plants has been extensively studied and is widely accepted as a plant-specific biosynthesis pathway [27]. It contributes to the synthesis of lignans, sinapate esters, stilbenes, and flavonoids [28]. Lignans are normally considered as plant-specific secondary metabolites [28]. *Phomopsis* sp. XP-8 can produce Pin and PDG in this study, similar to its host plant [15]. Successful identification of the phenylpropanoid pathway in *Phomopsis* sp. XP-8 might be due to this strain acquired essential genes from it host plants, since there is a possibility for the horizontal gene transfer between endophytes and host plants might happen during co-evolution [29].

The occurrence of phenylpropanoid pathway in *Phomopsis* sp. XP-8 was consisted with that found in other endophytic fungus that had capability to produce plant secondary metabolites. Some key enzymes involved in the pathway have been successfully used to construct genetically modified microorganisms to produce some plant second metabolites, such as resveratrol [30]. Besides of what was found in this study, the evidence of a phenylalanine pathway in *Alternaria* sp. MG1 (an endophytic fungus isolated from grape that can produce resveratrol) was shown to correlate with its resveratrol production [22]. Therefore, we suppose that the phenomena of the existence of thephenylpropanoid pathway in endophytic fungi producing plant metabolites of this pathway might be normal in nature. However, more evidence in genes and protein levels is still need to certify this hypothesis.

The failure of bioconversion from Pin to PDG in this study indicated that the biosynthesis pathway of Pin might be distinct from that of PDG in *Phomopsis* sp. XP-8. Furthermore, the production of PDG was only slightly affected, but the production of Pin was significantly decreased when the substrate was changed from Phe to Tyr, indicating that Pin may not only precursor of PDG in XP-8 cells. However, no definite evidence is available to show the bioconversion between Pin and PDG in microorganisms and plants.

Besides when Phe was used as substrate, biosynthesis of PDG and Pin was also found when tyrosine (Tyr) was used as the sole substrate in the *Phomopsis* sp. biosynthesis system. This indicates Tyr can act also as a benzene ring donor for the biosynthesis of Pin and PDG. This might be due to the presence of a TAL enzyme in *Phomopsis* sp. XP-8. According to that reported in plants, TAL converts tyrosine to *p*-coumaric acid [26]. *Phomopsis* sp. may also possess an enzyme with a similar activity.

In the current study, biosynthesis of PDG or Pin was not found when aliphatic amino acids and heterocyclic amino acids were used as substrates, even when 0.15 g/L glucose was present in the system. This indicates that aromatic amino acids might act as benzene ring donors for the biosynthesis of Pin and PDG. Whereas, the involvement of Leu in the biosynthesis of Pin and PDG in the presence of glucose might be due to metabolism of Leu to glucose through the gluconeogenic pathway, glucose can be metabolized to Phe through the shikimic acid pathway. The delayed peak time for the production of Pin and PDG via this route also correlates with the higher number of reaction steps in the pathway used for bioconversion of Pin and PDG from Leu.

Overall, the study identified and verified the capability of *Phomopsis* sp.XP-8 to biosynthesize Pin or PDG using substrates involved in the reported plant phenylpropanoid pathway. It also supported the existence of a phenylalanine pathway in microorganisms with similar reaction flows and related enzyme activities. In addition, the study also has novelty in illustrating the potential of *Phomopsis* sp. XP-8 to produce cinnamic acid, Pin, and PDG, and indicates that low C4H activity might be the bottleneck that determines the efficiency of the entire biosynthetic pathway. It is also interesting that Leu was found to have the ability to increase the bioconversion of Pin and PDG by *Phomopsis* sp.XP-8, likely through some kind of influence on the phenylpropanoid pathway.

## Supporting Information

S1 Table The occurrence time when the products reaching the highest value during the bioconversion using *Phomopsis* sp. XP-8 cells with glucose, leucine, and phenylpropanoid pathway intermediates as the substrate in the presence of glucose. (DOCX)

S2 Table The optimum concentration of substrates when the products reaching the highest value during the bioconversion using *Phomopsis* sp. XP-8 cells with glucose, leucine, and phenylpropanoid pathway intermediates as the substrate in the presence of glucose. (DOCX)

S3 Table The dry cell weight when the products reaching the highest value during the bioconversion using *Phomopsis* sp. XP-8 cells with glucose, leucine, and phenylpropanoid pathway intermediates as the substrate in the presence of glucose. (DOCX)

S1 File The original data points underlying all presented means and standard deviations. (XLSX)
