# Correction: Bioconversion of Pinoresinol Diglucoside and Pinoresinol from Substrates in the Phenylpropanoid Pathway by Resting Cells of *Phomopsis* sp.XP-8

**DOI:** 10.1371/journal.pone.0150129

**Published:** 2016-02-22

**Authors:** Yan Zhang, Junling Shi, Laping Liu, Zhenhong Gao, Jinxin Che, Dongyan Shao, Yanlin Liu

There are errors in the captions for Table 2, Fig 4 and Fig 5. Please find the corrected captions here.

**Table 2:** Production of Pin by *Phomopsis* sp. XP-8 cells using different amino acids in the absence of glucose. Values are the means of three replications and shown with standard deviation.

	Control	Amino acids (7.0 mmol/L) added in the control						
		Leu	Thr	Lys	Phe	Tyr	Trp	His
Dry cell weight (g/L)	1.02±0.12^d^	1.21±0.10^cd^	1.13 ±0.12^d^	1.31 ±0.14^bcd^	1.78 ±0.12^a^	1.64±0.12^ab^	1.56±0.14^abc^	1.82±0.14^a^
Pinoresinol (mg/L)	0^c^	0^c^	0^c^	0^c^	10.02±0.4^a^	2.92±0.22^b^	0^c^	0^c^

**Fig 4:**
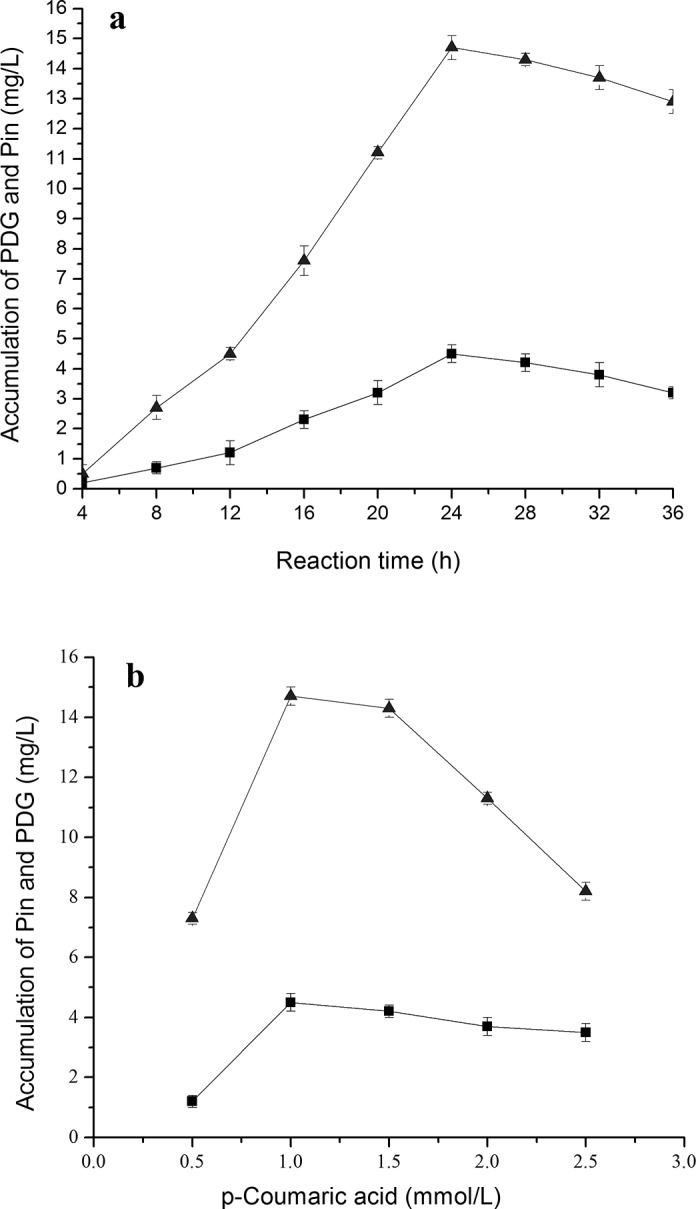
Effects of *p-*coumaric acid additions on PDG and Pin production. The signals in the figures indicate PDG (square), Pin (triangle). The used condition is 1.0 mmol/L *p*-coumaric acid additions (a) and reaction time of 24 h (b).

**Fig 5:**
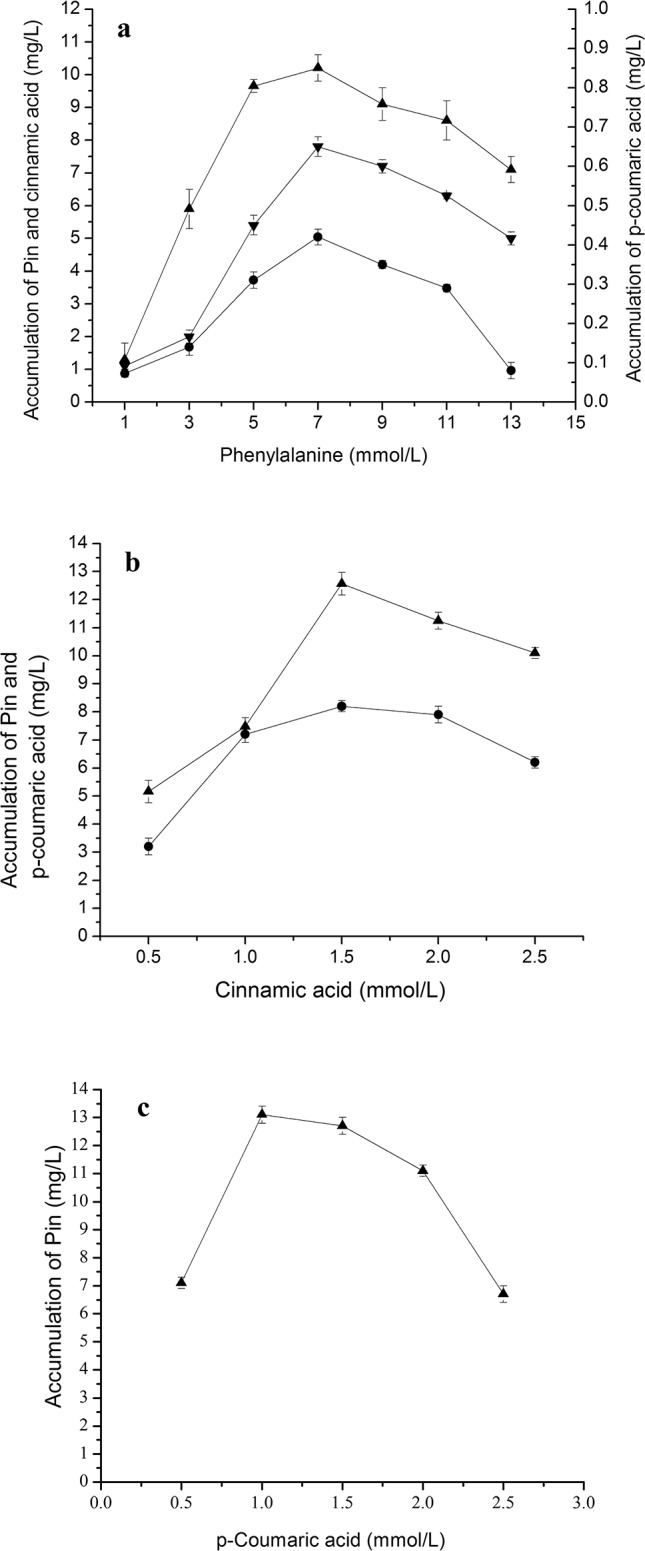
Bioconversion of Pin from Phe, cinnamic acid and *p-*coumaric acid. The reaction time was 40 h for phenylalanine in (a), 32 h forcinnamic acid in (b) and 24 h for *p*-coumaric acid in (c). The signals in the figures indicate cinnamic acid (downtriangle), *p*-coumaric acid (circle), Pin (uptriangle).
