# Supplementary material for: Bioconversion of Pinoresinol Diglucoside and Pinoresinol from Substrates in the Phenylpropanoid Pathway by Resting Cells of Phomopsis sp.XP-8
Source: PLoS One. 2015 Sep 2;10(9):e0137066. doi: 10.1371/journal.pone.0137066 (PMC4557914; doi:10.1371/journal.pone.0137066)
Supplement: S1 Table — (DOCX) [file pone.0137066.s001.docx]

**S1 Table. The occurrence time of the products reaching the highest value during the bioconversion using *Phomopsis* sp. XP-8 cells with glucose, leucine, and phenylpropanoid pathway intermediates as the substrate in the presence of glucose**

| products | Reaction time with different substrates added（h） | | | | | |
| --- | --- | --- | --- | --- | --- | --- |
|  | Glu | Leu | Phe | Tyr | Ca | pC |
| Phe | 32 | - | - | - | - | - |
| Ca | 32 | 40 | 32 | - | - | - |
| pC | 40 | 48 | 32 | 32 | 28 | - |
| Pin | 48 | 56 | 40 | 48 | 32 | 24 |
| PDG | 48 | 56 | 48 | 48 | 32 | 24 |

**Tabal S2.** **The optimum concentration of substrates when the products reaching the highest value during the bioconversion using *Phomopsis* sp. XP-8 cells with glucose, leucine, and phenylpropanoid pathway intermediates as the substrate in the presence of glucose**

| Products | The optimum concentration of substrates (mmol/L) | | | | | |
| --- | --- | --- | --- | --- | --- | --- |
|  | Glu | Leu | Phe | Tyr | Ca | pC |
| Phe | 111.11 | - | - | - | - | - |
| Ca | 111.11 | 13 | 7 | - | - | - |
| pC | 111.11 | 13 | 7 | 5 | 1.5 | - |
| Pin | 111.11 | 13 | 7 | 5 | 1.5 | 1 |
| PDG | 111.11 | 13 | 7 | 5 | 1.5 | 1 |

**Tabal S3. The dry cell weight when the products reaching the highest value during the bioconversion using *Phomopsis* sp. XP-8 cells with glucose, leucine, and phenylpropanoid pathway intermediates as the substrate in the presence of glucose**

| Products | The dry cell weight when added different substrates (g/L) | | | | | |
| --- | --- | --- | --- | --- | --- | --- |
|  | Glu | Leu | Phe | Tyr | Ca | pC |
| Phe | 1.96±0.15 | - | - | - | - | - |
| Ca | 1.96±0.15 | 1.69±0.14 | 1.66±0.14 | - | - | - |
| pC | 2.14±0.12 | 1.72±0.13 | 1.66±0.14 | 1.92±0.14 | 1.29±0.12 | - |
| Pin | 2.21±0.13 | 1.70±0.12 | 1.69±0.12 | 2.06±0.12 | 1.29±0.15 | 1.29±0.12 |
| PDG | 2.21±0.13 | 1.70±0.12 | 1.72±0.13 | 2.06±0.12 | 1.29±0.15 | 1.29±0.12 |
