# Supplementary material for: Bioconversion of Pinoresinol Diglucoside and Pinoresinol from Substrates in the Phenylpropanoid Pathway by Resting Cells of Phomopsis sp.XP-8
Source: PLoS One. 2015 Sep 2;10(9):e0137066. doi: 10.1371/journal.pone.0137066 (PMC4557914; doi:10.1371/journal.pone.0137066)
Supplement: S2 Table — (DOCX) [file pone.0137066.s002.docx]

**S2 Table.** **The optimum concentration of substrates when the products reaching the highest value during the bioconversion using *Phomopsis* sp. XP-8 cells with glucose, leucine, and phenylpropanoid pathway intermediates as the substrate in the presence of glucose**

| Products | The optimum concentration of substrates (mmol/L) | | | | | |
| --- | --- | --- | --- | --- | --- | --- |
|  | Glu | Leu | Phe | Tyr | Ca | pC |
| Phe | 111.11 | - | - | - | - | - |
| Ca | 111.11 | 13 | 7 | - | - | - |
| pC | 111.11 | 13 | 7 | 5 | 1.5 | - |
| Pin | 111.11 | 13 | 7 | 5 | 1.5 | 1 |
| PDG | 111.11 | 13 | 7 | 5 | 1.5 | 1 |
